# Analysis of the causes of spawning of large-scale, severe malarial epidemics and their rapid total extinction in western Provence, historically a highly endemic region of France (1745–1850)

**DOI:** 10.1186/1475-2875-13-72

**Published:** 2014-02-28

**Authors:** Emeline Roucaute, George Pichard, Eric Faure, Manuela Royer-Carenzi

**Affiliations:** 1 1 rue Edouard Millaud, Tarascon, 13150 France; 2 11 avenue du Parc Borely, 13008 Marseille, France; 3 Aix Marseille Université, CNRS, Centrale Marseille, I2M, UMR 7373, 13453 Marseille, France

**Keywords:** Malaria epidemics, *Plasmodium vivax*, Climatological data, Provence, Mediterranean area, Acquired immunity, Canal digging, Temperature variations, Altitude

## Abstract

**Background:**

The two main puzzles of this study are the onset and then sudden stopping of severe epidemics in western Provence (a highly malaria-endemic region of Mediterranean France) without any deliberate counter-measures and in the absence of significant population flux.

**Methods:**

Malaria epidemics during the period from 1745 to 1850 were analysed against temperature and rainfall records and several other potentially relevant factors.

**Results:**

Statistical analyses indicated that relatively high temperatures in early spring and in September/October, rainfall during the previous winter (principally December) and even from November to September and epidemics during the previous year could have played a decisive role in the emergence of these epidemics. Moreover, the epidemics were most likely not driven by other parameters (e.g., social, cultural, agricultural and geographical). Until 1776, very severe malarial epidemics affected large areas, whereas after this date, they were rarer and generally milder for local people and were due to canal digging activities. In the latter period, decreased rainfall in December, and more extreme and variable temperatures were observed. It is known that rainfall anomalies and temperature fluctuations may be detrimental to vector and parasite development.

**Conclusion:**

This study showed the particular characteristics of malaria in historical Provence. Contrary to the situation in most other Mediterranean areas, *Plasmodium falciparum* was most likely not involved (during the years with epidemics, the mean temperature during the months of July and August, among other factors, did not play a role) and the population had no protective mutation. The main parasite species was *Plasmodium vivax*, which was responsible for very severe diseases, but contrary to in northern Europe, it is likely that transmission occurred only during the period where outdoor sporogony was possible, and *P. vivax* sporogony was always feasible, even during colder summers. Possible key elements in the understanding of the course of malaria epidemics include changes in the virulence of *P. vivax* strains, the refractoriness of anophelines and/or the degree or efficiency of acquired immunity. This study could open new lines of investigation into the comprehension of the conditions of disappearance/emergence of severe malaria epidemics in highly endemic areas.

## Background

In the past, malaria was endemic and constituted a major health issue in marshy areas of France [1-3]. The last remaining foci of malaria in continental France (as Camargue) were extinguished from the end of the 1940s to the beginning of the 1950s [4-8]. In several endemic areas, such as Provence (southern France), intermittent fevers were so common that they were trivialized, hence usually not mentioned. Endemic zones were considered as unhealthy usually without any other comments; so, it is impossible to accurately estimate variation of the endemic rates. Contrarily, in the historical literature, epidemics, due to their severe health consequences, were generally mentioned with details.

The interest in studying western Provence lies in that in this previously highly endemic area, severe large-scale malaria epidemics occurred but then stopped abruptly in the 1770s, but not as the result of any deliberate intervention (e.g., drying of marshes, use of quinine, antilarval treatments or DDT) all of which had played determinant roles in other Mediterranean regions of Europe. Moreover, no real change in the socio-economic conditions or in agriculture or cattle breeding practices was observed during this period. In addition, in contrast to other Mediterranean areas, the population had not developed any known malaria-protective genetic trait. This situation represents a unique opportunity to investigate the causes and factors involved in the extinction of malaria epidemics in a highly endemic area. Moreover, another puzzle of this study is that severe epidemics were triggered in a region with a high malaria endemic rate and in the absence of any significant population flux. Malaria is suitable for spatial and temporal studies, and both the conditions for the emergence and decline of epidemics will be analysed in three different geographical areas for over a century (1745–1850). This work shows the complexity of the comprehension of the disappearance of malaria epidemics and could lay the foundation for assessing the risk of the emergence of severe epidemics in highly endemic areas.

## Methods

### Study area

**Figure 1:**
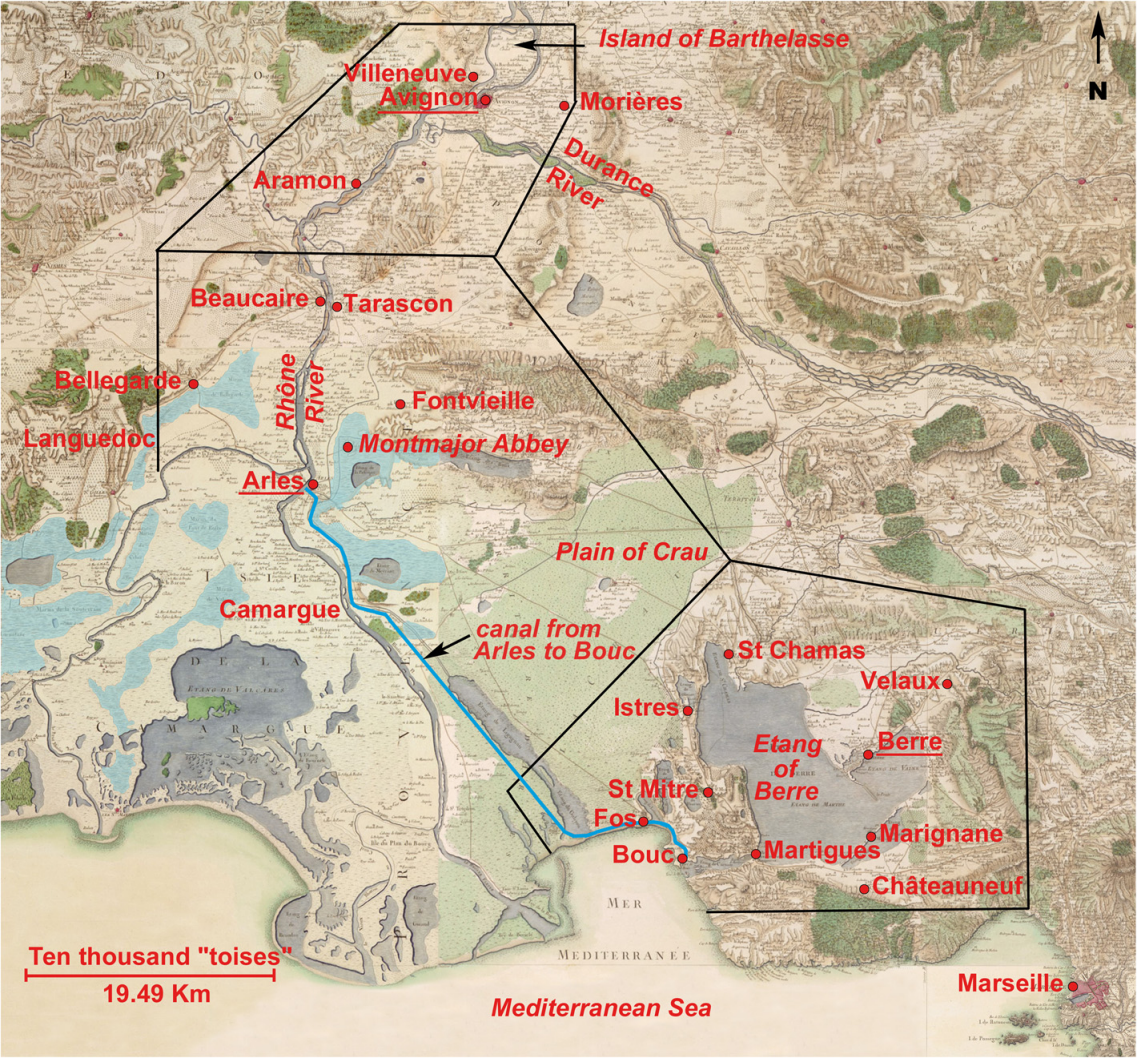
**Cassini’s map of the western Provence (1770–1776).** The geographic coordinates of this map are approximately 43.15 to 44.0 N and 4.20 to 5.30 W. The three studied areas have been surrounded by a black line; they carry the name of the underlined cities (i.e., Avignon, Arles and Berre). In the south-east, the area of Arles is bounded by the western arm of the Rhone. The positions of the towns and villages of the three studied areas mentioned in the text have been postponed. As meteorological data were measured in Marseille, this city has also been labeled. Data other than names of the towns or villages are in italics. Areas identified as wetlands by Cassini were stained in light blue, but the floodplains have not been indicated to not overload the figure. This map shows the great expanses of marsh and swamplands. Adapted from [9].

The study area is located in the south-east of France, a zone that spans less than 90 km. The base map used was one of Cassini’s [9] dating back to the second half of the 18^th^ century (Figure 1). However, it must be noted that these maps are rather imprecise and should be used primarily for information, e.g., to estimate the areas occupied by wetlands [10]. The studied area encompasses the historically western most part of Provence (i.e. the region between the Rhone River and the downstream portion of the Durance River), but for geographical, meteorological and epidemiological reasons surrounding areas are included (these are the region of Avignon situated at the confluence of the Rhone and Durance Rivers and municipalities on the left bank of the Rhone River). Moreover, the names of the communes affected by epidemics were not always mentioned and could be replaced by terms such as “in Arles and its region”, “around Avignon” or “in the edge of the Etang of Berre”. Thus, it seemed most appropriate to subdivide the studied zone into three areas. Two of the areas were named for their principal urban area (Avignon and Arles), whereas the third area (Berre) consisted of villages around the “Etang of Berre” (Figure 1, Additional file 1). These three zones are located on a broad north–south transect. The studied area has a Mediterranean climate characterized by warm, dry summers and mild, wet winters. Moreover, the landscape consisted of wet areas (e.g., coastal lagoons, marshes) and dry areas (e.g., agricultural zones, Mediterranean scrubland, forests).

In each region, the relative percentage of wetlands is difficult to estimate due, among other reasons, to variations over time. In the Avignon area, the marshy lands were less numerous than in the two other regions; however, an encyclopedia of the late 18^th^ century mentioned that wetlands were still covering large areas in the south of the city [11]. Moreover, the risks of floods due to the Rhone River were high in the lowlands. In the Arles area, the altitude was mostly close to the sea level and ponds occupied a large area. Draining works were carried out in the 17^th^ century, but due to willful destruction, flooding or non-maintenance of works, the region remained very marshy [12]. During the French Revolution (approximately 1792), the marshes and ponds around Arles (principally those of Arles *sensu stricto* and of the Baux at the east of Montmajor Abbey) had almost recovered their areas of yesteryear [13]. The area of these wetlands was estimated at 313 km^2^, of which the 60 km^2^ of the Baux [14,15] formed a network impenetrable enough to serve as a refuge for refractory priests during the First Empire (1804–1814) [16]. In a report of 1817, it is noted that there were still immense marshes or ponds near Arles [17]. Moreover, due to their low altitude, a great part of this area was inundated by floods of the River Rhone. The draining of these marshes was principally accomplished in 1843–50 [12,18]. In the region of the Etang of Berre, several aquatic areas could be found, including the eponymous pond, which is a large lagoon communicating with the Mediterranean Sea, stagnant marshes and ponds and other communications with freshwater flows or with the Etang of Berre [19]. In 1809, a physician mentioned that most agglomerations were surrounded by several marshes [20]. In addition, in this area, which was not subject to the vagaries of the Rhone, there could be flooding covering wide ranges of the relatively flat and low-altitude region. Moreover, here as elsewhere, in most of the towns of Provence, there was a great deal of stagnant water. For example, in 1810, a physician said that in Martigues (Berre area) there was mud extracted from the canals and water rotted in the vicinity of houses, smelly gas emanated from muddy brooks and ruts caused by cartwheels and there was no slope for drainage of water and liquid waste [20]. The anopheline breeding places could, therefore, be numerous owing to physico-chemical conditions and to the permanence of water.

Based solely on geographical and hydrological criteria, the studied area could exhibit a high malaria risk - a point that has been confirmed by contemporary physicians. In western Provence during the second half of the 18^th^ century, the data are too imprecise to quantify the endemic rate accurately; however, extensive analysis of historical documents from physicians evidenced that the worst region was Berre, suggesting the highest malaria endemic rate [21-24]. The endemic level can be estimated intermediate in Arles and lower in the region of Avignon even if intermittent fevers raged relatively frequently [25]. However, in a single area, there could be great disparity in endemicity, e.g., inside the towns’ walls of both the Avignon and Arles areas, the pattern was generally always epidemic.

Although meteorological data have been obtained for Marseille, the most populous city in Provence, this town was not integrated into this analysis due to its very complex epidemic constitution [26]. Moreover, the Camargue, which covers the Rhone River Delta and is still currently the main wetland area of Provence, was not included in the epidemical analyses; indeed, this area was almost totally uninhabited [27], with a population density of around two inhabitants per km^2^ in the 1760s [28] and so, during the studied period, true epidemics were rarely mentioned by witness authors. However, data which might help to understand the endemo-epidemic situation in Provence were incorporated into this study.

### Epidemiological data

Malaria diagnosis by identification of *Plasmodium* species using microscopic examination of blood films dates only from the end of the 19^th^ century and, therefore, before this period historical retrospective diagnosis is always difficult [29]. However, if data in the ancient texts are sufficiently pertinent, exhaustive analyses can afford to have very strong arguments in favour of the involvement of malaria, even for very old times [30]. Up until the end of the 19^th^ century, both residents and visitors to Provence used various names to refer to what is now called malaria. They described “marsh fevers” using terms highly suggestive of malaria infection, such as “intermittent fevers” (i.e., fevers with alternate fits of heat), “tertian or quartan fevers” or “fevers with bouts” [31], whereas common people used vaguer terms as “the shaking or shivering fit” [32]. Sometimes, only the word “fevers” was mentioned, but this term again generally referred to malaria, as previously stated [30,33]. In addition, mentions of successive alternating hot, cold, and sweating stages are useful, but the periodicity and intermittency of the fever fits are the most notable features permitting retrospective diagnosis. Analyses of medical texts of Provence added to other writings demonstrated that in this area, among intermittent fevers, tertian fevers predominated and quartan fevers were rarer [25]. Moreover, tertian fevers may have variably qualified as benign, malignant, pernicious, etc. *Plasmodium vivax* and *Plasmodium ovale* were generally supposed to cause the so-called benign form of tertian fever and *P. falciparum* the pernicious form of tertian fever, whereas *Plasmodium malariae* caused quartan fevers. In Provence, the implication of *P. ovale* was highly improbable as its current range was limited to West Africa and East Asia. The involvement of *P. malariae* is indisputable, but there is no reason to insist on this species because it did not seem directly involved in the major epidemics observed in Provence. It is just possible to report that this species persists for a long time in the organism with many relapses and so induces a general weakening of the infected individuals and also that the periodicity of the fevers was most frequently observed during winter, the season when the background noise of other diseases such as gastroenteritis was lower.

**Figure 2:**
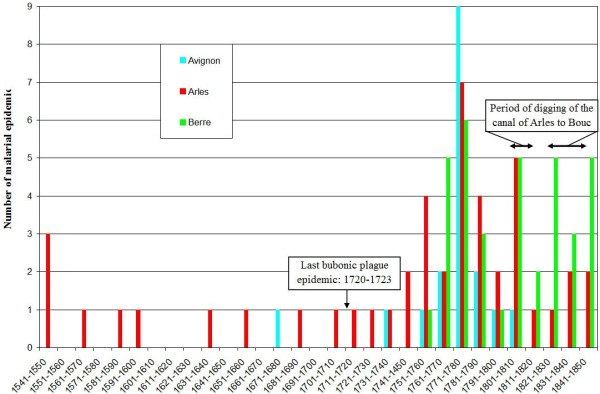
**Number of malaria epidemics in western Provence.** The number was given by area and by decade from 1541 to 1850.

In malarious areas of western Provence, malaria was a component of the environment; thus, no great attention was paid to the endemic forms of this disease. Every inhabitant was permanently infected from the day he was born to the day he died, and survival depended principally on acquired immunity [34] or on the possibility of leaving the region for a healthier one. Moreover, cases of intermittent fevers were mentioned more frequently when they affected wealthy persons than poor; the poor and vulnerable living in the unhealthiest areas were likely the most affected by malaria rather than the wealthy. In addition, the number of deaths per year for each village was not always known in the earliest periods, and even when it was, the cause of death was not indicated in death registries. However, contrary to malaria endemicity, malaria epidemic episodes were relatively frequently listed and described with proportionately greater numbers of details. Records on malaria epidemics have been collected by Roucaute from 1541 to 1850, principally using historical documents [25,31] (Figure 2) (see Additional file 1 for the period 1745–1850). Biases are inherent in this type of research that ranges across several centuries, rendering statistical analysis impossible, due, among other factors, to terminology and the scarcity of sources for the earliest periods, as well as due to the fact that the number of epidemics is still lower than the reality because the sources are discontinuous over space and time. Regardless, this analysis suggested a relatively low frequency of epidemics from 1541 to 1750, followed by the worst period (first part of the second half of the 18^th^ century), and then a decline starting in the end of the 1770s and continuing through the next century (Figure 2). The period from the mid-18^th^ century to the mid-19^th^ century corresponds to the most recent time period analysed by Roucaute [25,31], which is the time frame with theoretically more documents with more details. Moreover, in this region, instrumental meteorological records are available since 1745. However, the main question concerns the criteria defining a disease episode as a malaria epidemic (i.e., an epidemic episode for which the main causative agent should be a *Plasmodium*). Much attention was paid to the origin of the authors who mentioned epidemics; indeed, as suggested by Brès [35], it would be an error to consider as an epidemic a hitherto unrecognized endemic situation or a simple seasonal increase in the incidence of the disease. Foreigners in the studied area, whether physicians or not, may have been surprised by the number of sick individuals and may have interpreted the situation as an epidemic, whereas the numbers only represented the usual victims of endemic malaria. In addition, mentions of epidemics of remittent fevers have been frequently found in the texts [19,25]. With this type of fever, patients remained feverish with significant temperature variations over 24 hours (more than 1°C). In contrast, with intermittent fevers, recurring febrile episodes were separated by times of normal temperature. Several authors since the last part of the 19^th^ century have considered remittent fevers as a particular form of malaria. In some areas, remittent fevers might almost always be the first manifestation of malaria because they transform over time into intermittent fevers; in addition, some of these remittent fevers resemble continuous fevers, but with fits similar to those of simple tertian fevers [2,36]. In this study, if a significant number of mentions of intermittent fevers was not associated with these epidemics, these have not been included in the dataset. A similar approach has been taken with regard to malignant or pernicious fevers. This approach shows that it is necessary to critically consider all the terms mentioned in the texts because historical data on diseases and medical events are difficult to analyse, especially at times when the malaria pathogen was not known. However, even if other causes of mortality and morbidity contributed to the deterioration of the health situation, Gayte and Nicoli [18] have shown that, at least in Provence, precise symptomatic tables permit to incriminate malaria in a reliable manner.

Finally, in this study of the 1745–1850 period, an epidemic was considered as corresponding to a malaria epidemic if the following conditions were satisfied: 1) intermittent fevers affecting more people than usual; 2) the episode was mentioned as an epidemic by at least a physician or was considered as similar to another epidemic of the same period and in the same area, and in which the notion of intermittence of the fevers was considered as unambiguous as mentioned by a physician or several non-medical authors; 3) in an endemic area, an epidemic corresponded to an important increase in specific morbidity and/or mortality; and 4) in an area with very few or no endemic fevers (e.g., in town centres or in high altitude spots), a significant number of people were affected by intermittent fevers. Precise data concerning malaria epidemics in Provence since the mid 18^th^ century can be found in various works of synthesis of the 20^th^ century made by historians, physicians and pharmacists who made critical analyses [18,19,25,31,37]. However, it is very difficult to estimate the morbidity and mortality rates during malaria epidemics in Provence, even when data were provided by primary sources, and even if the witnesses were of good faith and rigorous, these latter should always be used with caution. For example, patients in hospitals might come from more or less distant areas and they could be counted in the number of fatalities of the town after their death. To estimate the mortality rate, it is necessary to know the number of inhabitants, or this would be inaccurate. Second, as with plague, the standard advice of people facing great malarial epidemics was *cito, longe, tarde,* which translates from Latin as “leave quickly, go far away and come back slowly”. The data may be more accurate in villages than in cities because there was no hospital, fewer people were unaccounted for because they were needy and, generally, as most of the people were not wealthy, few individuals were fleeing the place during the epidemic. In addition, when the number of deaths was given during an epidemic, it may represent persons dying from all causes. However, despite all of these restrictions, historical data indicate that malaria epidemics of the period 1745–1776 had very high morbidity and mortality rates in urban populations. Two examples, provided from accurate records of physicians or surgeons and evidenced by the intensity of the morbidity rate, are mentioned below. During the summer of 1766, intermittent fevers were so prevalent that they struck down almost the entire population of Berre and some remained ill for several months to a year [22]. During the autumn of the same year, 98.3% of the inhabitants of a village in the south of the Etang of Berre suffered from intermittent fevers [25].

### Meteorological and miscellaneous non-epidemiological data

Although long series of instrumental climatologic data from the middle of the 18^th^ century are very rare, databases of homogeneous daily series of temperature and rainfall over a city very close to the studied area (Marseille) (see Figure 1) are available for this purpose. These data were compiled by Pichard and Roucaute [38-40]. The data cover the periods starting in 1745 and 1748 for temperature and rainfall, respectively, and, therefore, allow the analysis of the period of the epidemic breakpoint dated in the 1770s. According to the periods, recordings were made in two different stations (but close to each other: Catelin from 1748 to 1787 and the Observatory of the Accouters from 1766 to 1863) and in dissimilar conditions (e.g., two or more daily temperature measurements). However, as the measurements overlap during certain periods, homogenization of the data was feasible. Moreover, the temperature data for Marseille was lost for the periods 1793–1805 and 1815–1822, but Guerin’s database with recorded temperatures at Avignon city is available from 1802 to 1840. Next, the common periods (1806 to 1814 and 1823 to 1840) were used to calibrate a model to deduce most of the missing temperatures that would have been recorded in Marseille, given those measured in Avignon. Hence, no data can been found in the region for the period 1793 to 1801. Consequently, the missing values were merely replaced by the associated monthly means (Additional file 2). The rainfall data for Marseille come also from two different databases. To homogenize the data, data from the shared period (1766 to 1787) of both databases was used to calibrate a model to deduce the rainfall data used in this article (Additional file 2). In this study, even if daily rainfall and mean temperature data were available for almost all the years, usually only monthly rainfalls and mean temperatures monthly data were used.

Several socio-economic variables known to influence the course of malaria were also analysed, this is mainly social events, data concerning agriculture (as cattle practice [41], freeze of olive-trees [42-44], rice cultivation [41,45]) and civil engineering works [25,37,46,47] (Additional file 1). Recent studies have suggested that malaria was basically a sociological disease for which household size and housing standard appeared to correlate very closely over a long interval with the decline in malaria cases [48-50]. However, in Provence, during the period studied, despite extensive research, there were too many temporal and spatial gaps to be able to carry out correctly this type of statistical analysis. Data and references concerning past distribution of anophelines in Provence can be found in Ponçon’s thesis [51]. Current data, mainly from Camargue, has been collected by the Didier Fontenille’s laboratory in Montpellier, France [51-56].

### Statistical analysis

Homogenized monthly total rainfall and mean temperature for Marseille were analysed to estimate the influence of weather conditions on malaria epidemics. Note that data from November and December of the previous year have been used. Similarly, various other factors linked to climate (e.g., drought) that could be potentially correlated with epidemics were also analysed. The presence of epidemics during the previous year was also considered as an explanatory variable. Statistical analysis was performed with the R freeware [57] and p-values < 0.10 were considered significant.

First, the effect of several factors on epidemics was analysed using classical tests: the Chi-squared test of independence for binary factors (e.g., presence *vs.* absence of floods, epidemics during the previous year), a generalized linear model with a Poisson error distribution for discretized factors (e.g., frequency of floods or epidemics in the previous year) and either Student’s *t-*test for sample sizes of 30 or more per group or Wilcoxon rank-sum test for small samples for continuous factors (temperature and rainfall). A simple linear model was used to test the correlation between two continuous factors.

Next, multivariate analyses were performed to summarize the data. Note that principal components are interpreted thanks to the angles between the arrows pointing for any quantitative variable and the principal components since the cosinus values equal the correlation coefficients. Year’s coordinates on the principal components were computed according to meteorological data only. The binary epidemic factor was used as a supplementary variable, and the associated ellipses were added in order to illustrate data consistence. The smaller is the ellipse, the more homogeneous are meteorological variables for the associated years. Note that ellipses should contain 68% of the data under Gaussian bivariate distribution.

Finally, time series theory has been used to model temperature before the epidemic breakpoint in order to compare the forecasts with the observed data. The best AutoRegressive Integrated Moving Average (ARIMA) model was seeked by maximizing AIC criterion [58]. The obtained white noise model was validated with Ljung-Box test and residuals normality was tested using Shapiro-Wilk and Jarque-Bera tests. In this case, predictions are all the mean value and twice the standard deviation (estimated by maximum likelihood) provides the fluctuation interval.

## Results and discussion

### Data and analyses concerning the malaria endemicity in western Provence

#### Analysis of the data concerning the intensity and evolution of malaria endemicity

The precise timing of the malarial endemic recession, and its declining impact on mortality, certainly seems to have varied from one marshland area to another. Gayte and Nicoli [18] noted that the decline of endemic malaria in all the regions most likely began in the late 18^th^ century and continued gradually decreasing until 1850, a period when the disease became relatively rare, if Camargue was excluded. Furthermore, as the latter authors emphasized, scientific and rational methods of fighting malaria began only around 1900 in this region. However, the decline in endemicity was weaker than some authors have assumed. There was often a tendency to forget over time the severity of malaria endemicity or even of epidemic episodes; thus, when possible, the most contemporary documents of the epidemic episode in question must be used. Examples of the underestimation of the malarial risk are numerous and not unique to Provence; in the 17-18^th^ centuries, parishes around London were invariably described as “healthy” and “pleasant” but registered high burial surpluses [59]. Some authors, who are rarely physicians, could have denied that the health situation was deleterious during the period in which they lived for several reasons [15,46,60]. Some of them are economic, as they wanted that their home regions to be considered attractive. It was, therefore, necessary to critically analyse the largest number of contemporary documents for a given period. Moreover, it is only in comparison with the intense malaria in some areas that other regions appeared extremely healthy. Accurate analyses have shown that the number of potential malarial reservoirs remained high in the early 19^th^ century, even in the Avignon area, people were annually infected by intermittent fevers, although malignant and fatal fevers had become very rare [61]. Moreover, in the area of Arles of the mid-19^th^ century (and in Camargue later), the number of individuals with acquired immunity was most likely still high [62,63] and the life expectancy was still very low [64]. However, the decreasing malaria pressure was real. In the 1830s, in the area of Arles, physicians reported a “*strange change in the medical constitution*”, with intermittent fevers being less frequent and less intense than in the past [65,66]. It was also noted as surprising that in the late 1820s to early 1830s, the cleaning of channels in both Arles and Tarascon - an operation dreaded by the population that could only be performed in summer due to the low water levels - was performed without manifestation of intermittent fevers, contrary to parts of Berre [67]. In the first half of the 19^th^ century, the endemic pressure exerted by malaria also continued to decline in the area of Berre but did so more slowly. In 1810, a physician of this zone reported that whereas many diseases other than malaria could end with intermittent fevers, these fevers could either precede these same diseases or accompany them [20]. Moreover, a study concerning Berre during the years 1839–1841 showed that intermittent fevers were still very common, but were less dangerous than observed previously [68]. These fevers were less severe and non-lethal, and they could be cured by quinine; this improvement was attributed to the drying of many marshes that existed in the vicinity of Berre. Indirect data corroborate this decrease in endemic malaria; e.g., from 1800 to 1841, the population of Fos (in the area of Berre) almost never ceased to grow at a high rate and the number of inhabitants more than doubled, even if this trend was partly due to population flows outside the town or country [69]. Two other elements also suggest that the parasite was well on the decline. Mortality crises, even if they were rare, did occur but could not stop population growth significantly, despite that during this period, a canal was constructed from Arles to Bouc and there was population flux from highly malarious areas of Italy settling around the Etang of Berre [69]. Moreover, the relatively more frequent mentions of the number of quartan fevers during winter, and even sometimes during summer since the first half of the 19^th^ century [25], most likely correlated with a decrease in the rate of *P. vivax* endemicity.

#### Temperatures in Provence and *Plasmodium* sporogony

Detailed analyses of climatic factors will be studied later, but at this stage, the possibility of outdoor sporogony in Provence needs to be addressed. Temperature is critical to the spread of malaria as it plays a role in the development of both anophelines and *Plasmodium.* The impact of temperature on the sporogonic development of *Plasmodium* parasites within insect vectors is well documented. Grassi and MacDonald estimated threshold temperatures under which the sporogonic development is not completed, but these figures are not precise thresholds and may vary from between 14.5-17°C for *P. vivax* and 18-19°C for *P. falciparum*; the sporogony of *P. malariae* begins at a similar temperature as *P. falciparum,* but requires much longer periods [70,71].

**Figure 3:**
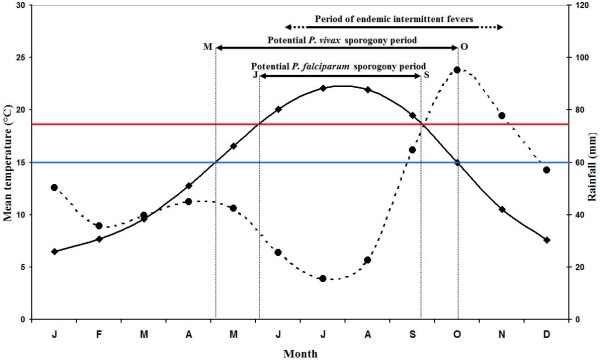
**Marseille climate diagram for the 1745–1850 period.** Average temperatures (solid line) and precipitation (broken line). The horizontal blue and red lines indicate minimal temperatures required for *P. vivax* (15°C) and *P. falciparum* (18°C) development, respectively. These figures are not precise thresholds, but they illustrate a climate above which temperatures are generally favourable for transmission.

**Figure 4:**
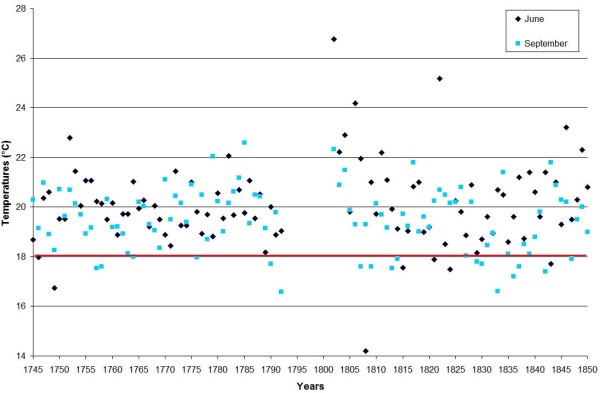
**Mean temperatures in June and September at Marseille.** The horizontal red line indicates minimal temperature required for *P. falciparum* (18°C) development.

Figure 3 shows that in Marseille from 1745 to 1850, during the months from May to September, the mean temperatures were compatible with the minimal temperature of development required for *P. vivax* (16.5, 20.1, 22.0, 21.9 and 19.5°C for May, June, July, August and September, respectively). The lowest average temperatures recorded in July and August were respectively 19.1°C and 14.4°C (in 1843); for all other years, they were above 19.4°C and thereby sporogony of both *P. vivax* and *P. falciparum* was theoretically always possible during these two months. Figure 4 analysed the mean temperatures in June and September. The sporogony of *P. vivax* was theoretically always possible during these two months with the exception of June 1808, whereas those of *P. falciparum* was impossible during eight of the summer months between 1745 and 1792, and in sixteen summer months in the course of the first half of the 18^th^ century. During the studied period, the mean temperature dropped below 15°C during only three months of May. The development of *P. vivax* parasites is compromised below 15°C [72], and during the month of October, the mean temperature was lower than 15°C in 45% of the years. In addition, analyses of mean daily temperatures show that the sporogony stopped after 15–20 days during the hottest October months, whereas it ceased rapidly in the initial days during the coldest months. Moreover, when taking into account both the mosquito and parasite life-history traits to temperature, the minimal temperature could be higher than those for sporogony alone, e.g., for *P. vivax,* it could be from 17°C to 20°C [73]. Therefore, if the complete development of *P. vivax* requires approximately 30 days at 18°C and stopped below 17°C [74], which is generally the case in Provence, sporogony could start only after mid-May, and could no longer come to term if started after mid-September. The period of transmission of *P. vivax* was thus from mid-June to September, which was compatible with the data transmitted by physicians regarding the endemic conditions - i.e., in Provence in the studied period, as spring fevers are rare, endemic intermittent fevers generally appeared in June, rarely in May; their numbers were increasing in July, and they reached their maximum growth in August-September [25,68,69]. Relapses could be observed in November and December. This seasonality was also mentioned when the endemic declined as in the 1830s in Arles [66].

In western Provence, despite the fact that the period studied is during the Little Ice Age (LIA), the average temperature from June to September was generally approximately 21°C, allowing for six potential generations for *P. vivax* and for five or less during the worst years [75]. In addition, according to the model of Mordecai *et al.*[73], optimal malaria transmission occurs at mean monthly temperature of 25°C, whereas the transmission decreases dramatically at temperatures >28°C; in western Provence, however, daily mean temperatures higher than 28°C were relatively rare. The climate diagram indicates that theoretical favourable temperature conditions can be generally found between June and September for both *P. falciparum* and *P. malariae* (Figures 3 and 4). For most of the years (76%), the mean temperature taken month to month was always higher or equal to 18°C. Considering that the complete sporogony for *P. falciparum* species needs around one month at 20°C and 19 days at 22°C [74] - and also the daily temperature in the beginning of June and the end of September - during summer, four potential generations could be allowed, but this number could be reduced to two or less during the coldest years. The cycle of *P. malariae* is longer, requiring 36 days at 20°C [74]. Thus, taking into account the ecology of the anophelines, the number of potential generations by year was null or one. This species can only be transmitted by long-lived vectors, which explain why it is less prevalent than *P. vivax*. Based only on the criterion of temperature, in western Provence the transmission of *P. vivax, P. falciparum* and *P. malariae* could be considered as stable, unstable and very unstable, respectively. However, as parameters other than temperature are needed to allow transmission of *Plasmodium* to human by anopheline bite (e.g., moisture and breeding places), outdoor transmission even for *P. vivax* would have been relatively low in some years.

#### Indirect mortality, life expectancy and sink populations in Provencal malaria endemic areas

Several analyses have shown that in historical Europe and in other continents where endemic malaria conditions occurred, the malaria-related mortality was an indirect result of the effects of malaria infection combined with other infections and conditions [33,59,76-79]. Indeed, after the reduction of malaria transmission, reductions in total mortality rates were observed to be several times greater than the malaria-related death rates estimated prior to the interventions [80,81]. Some of this discrepancy may be due to failures to detect or account for all the deaths for which malaria was an apparent cause. Due to the lack of intermittent temperature monitoring during for example, primary infection, multiple infections by various *Plasmodium* agents and co-infections with other infectious agents, malaria was not diagnosed. In Provence, most of the excess deaths were associated with, but not directly due to, the presence of malaria [24,25]. These deaths may be due to the predisposing effects of this infection to cause death from other conditions, such as gastro-intestinal disorders [33]. Moreover, following the summer-autumnal infectious period, numerous people who had suffered from severe malaria were undermined during the following winter and, therefore, could be more susceptible to other diseases. In addition, many people were still suffering from malaria during the winter. Detailed contemporary studies have demonstrated, as in the case of the gastro-intestinal diseases, those death rates from respiratory diseases alone are much lower than the death rates when malaria is also present [33]. In historical studies, the malaria-specific mortality rates do not represent an accurate index. First, the cause is rarely known, as was the case in Provence during the studied period. Second, contrary to the total rates, malaria mortality rates do not have the added advantage of reflecting both the direct and indirect effects of the disease. Thus, only indirect methods for estimating the malarial effects on mortality, such as comparing overall mortality levels (most simply by crude death rates), are useful. These indirect methods have been chosen by professional malariologists [33,59,77]; it is the same with this study but only for epidemic episodes.

Recurrent infections with any species of malarial parasite can be so debilitating that life expectancy can be reduced to half or less of that in a contemporary malaria-free and otherwise salubrious environment [82,83]. In the second half of the 18^th^ century, in all the higher malarious regions of Provence (banks of the Durance and Rhone Rivers, areas of Arles and Berre), the estimated average life expectancy (17–19 years for the lower values) were from 10 to 20 years lower than those observed in safe areas [21,24,84,85]. In the 19^th^ century, even if the period of the great epidemics of malaria had ceased, the pressure exerted by endemic malaria was still important. For example, in the 1850s, an author observed that the mean life expectancy was 21 years in the district of Arles versus 38 years in a town for which intermittent fevers were almost unknown whereas they were very common in Arles [64]. However, despite the risks, malarial areas have often been very attractive to inhabitants [32,82,86,87] due to the richness of the marsh soil, sometimes being the only places where the poor – generally, but not necessarily, men – could find work. Moreover, in these areas, people were resigned and fatalistic, and fevers constituted an intrinsic part of their lives [86,88,89].

In severely malarial endemic districts, direct and indirect mortality summed to a substantial reduction in the fecundity of a population due to abortions, greatly contributing to depopulation due to malaria [81]. In these areas, populations acted as a “sink”. This concept is drawn from studies of animal populations in which the latter were found in an environment that did not provide suitable conditions for reproduction or survival of individuals, such that “sink” populations cannot sustain themselves and have to be sustained by immigration. For example, with regard to human beings, sink populations have been described in past Italian and English marsh areas [33,87,90,91]. Similarly, since the 18^th^ century, populations with these characteristics have also been described in various past malarious areas of France [1] including those of Provence [92,93]. In endemic areas of Provence, frequently in the second half of the 18^th^ century and even in the beginning of the 19^th^ century, the number of deaths significantly exceeded births [19,25,37,68,94,95].

### Complex pattern of acquired immunity

After repeated bouts of malaria in a relatively short period of time, an individual develops a non-sterilizing immunity that does not prevent parasites from developing and circulating in the blood after a new inoculation, but does generally suppress the development of severe clinical symptoms [96]. Within an indigenous population, this partial protective immunity is generally acquired during early childhood, although at the expense of a heavy tribute in this age group. Subsequently, mortality rates in older children, teenagers and adults are far lower, except in pregnant women. However, acquiring such a state of premunition against malaria is a short-lived process, requiring continuous reinfections under high endemic conditions for maintenance. The acquisition of antiparasitic immunity, which is generally strain-specific [97], requires that an individual be habitually subjected to a succession of inoculations of parasites that are genetically and antigenically distinct. Only when a sufficiently wide spectrum of such parasite strains has been experienced is effective immunity achieved against all the parasites within a locality where an infection is endemic [81].

The arguments in favour of relative immunity in the indigenous population of Provence are numerous. For example, in some epidemics, only foreigners to the region were affected by malaria (e.g., during the digging of the canal from Arles to Bouc); in other epidemics, almost all the children suffered whereas adults were spared [98]. The inhabitants of villages that were located at higher altitudes than those of surrounding high endemic areas were particularly susceptible to malaria, which apparently always triggered an epidemic form [25,99,100]. The urban wealthy could be more sensitive in terms of morbidity to malaria. In the rice-fields of Camargue, foreign workers were more afflicted by fevers than were natives, even those coming from highly malarious areas such as Italy in the 19^th^ century. This last example shows that, at least in this case, the acquired immunity seems to be strain-specific. Moreover, this information suggests that, in Provence, foreigners knew the risks they took in coming to work in swampy areas and that they were more susceptible to malaria than the natives [25]. Similarly, during the last part of the LIA, in marshy areas of England, the frequency of malaria attacks and their highest incidence among the non-immune sector of the population (children and foreigners) reflected the high level of endemic malaria [59].

Intuitively and empirically, the concept of premunition was understood by early observers, such as in 1810 by a physician in the region of Berre [20]. This author also mentioned people living with impunity in the midst of swamps, whereas others living in very healthy areas suffered from intermittent fevers, even if they were wealthy. However, the “partial” resistance observed in malarious areas, related to the fact that the duration of the incubation period and the site of infection were unknown, made it very difficult to understand the origins of malaria fevers.

#### Probable rarity of indoor malaria transmission outside the outdoor sporogony period and spread of malaria by outdoor seasonal workers

Due to temperature conditions in northern countries or during a cold period, such as the LIA, malaria transmission could continue as it mainly occurred in indoor conditions due to the transmission of sporozoites throughout the winter by semiactive hibernating mosquitoes; thus, malaria could be relatively independent of outdoor temperature conditions [101]. An indoor transmission cycle in the Netherlands during the beginning of the 20^th^ century was described in detail by Swellengrebel and de Buck [29]. This mechanism of infection would also play a significant role in the marshy areas of England during the LIA [59]. Moreover, a retrospective analysis of endemic malaria in Finland during the 19^th^ century suggests that it existed only as an “indoor” disease [101]. Despite the severity of the temperatures, malaria could be still transmitted by *Anopheles maculipennis s.l.* mosquitoes – generally *Anopheles atroparvus*, but *Anopheles messeae* could also have played the major role, as in Finland – which survived by over-wintering in human habitations and cattle shelters [59,101-103]. Indoor transmission could explain that in Europe, during the LIA, malaria appeared, if not to move further northwards, at least to remain in the high northern areas [101,104]. In the summer, the parasites were dormant, which was crucial for maintaining the *Plasmodium* until the presence of the next generation of anopheline females. Moreover, when it is thermally possible, indoor transmission can coexist with outdoor summer transmission.

However, in Provence, even during the LIA, the climate was relatively mild without sustainable extreme temperatures during summer. Based only on thermal conditions and as shown in Figure 3, outdoor transmission, at least for *P. vivax*, could be theoretically possible every year. This trend can explain the seasonality of malaria in this area. Generally, endemic malaria was only observed during approximately three months (from mid-summer to autumn) [25,105], whereas spring intermittent fevers were usually rare and benign [106]. Moreover, during epidemics of malaria, the peak was almost always observed in the summer-autumn period [25]. All these elements taken together suggest that indoor transmission of sporozoites throughout the winter by semiactive hibernating mosquitoes was rare, if not excluded. Moreover, temporal data suggest that indoor transmission could occur, but mainly when outside temperatures allowed outdoor transmissions.

Malaria transmission is facilitated when large numbers of people sleep outdoors during hot weather, or sleep in houses that have no protection against mosquito invasion. Farm workers often slept out in the fields during the harvest, where they were very vulnerable to mosquito bites. The vulnerability of farm workers to malaria infection during the harvest has frequently been noted in Italy and other Mediterranean countries and, indeed, all over the world, wherever malaria occurs [33]. However, while in Italy peasants suffered frequently from pernicious malaria due to *P. falciparum*, in Provence, they were usually affected by relatively benign tertian fevers due to *P. vivax*. In western Provence, during the beginning of the 19^th^ century, a physician reported that many farmers had been observed to suffer from fevers after sleeping outdoors to guard the harvest before threshing [20]. In addition, the seasonal workers who performed harvest and other fieldwork in the regions of Arles and of Berre were accused, probably rightly, of introducing malaria on their return to safe areas, including to middle altitude villages [85,107,108]. Moreover, each year in late July, 3,000 to 4,000 poor people around Arles would steal during two months in the salt marshes of Camargue, themselves infected with fevers, and upon their return would contaminate people in Arles [27,109]. These nocturnal outdoor infections played an important role in the local diffusion of malaria.

Although historical data rarely give indications on this subject, generally in western Provence, the incubation period was short (one to three weeks) with a series of relapses at more or less short intervals, which corresponds, according to one of the classifications of Type I *P. vivax* strains principally found in tropical regions [110]. The two other types were Type II, with a short incubation period (two to three weeks) but a prolonged period (7–13 months) of latency between the primary attack and the first relapse or series of relapses at short intervals, and Type III, for which the primary infection occurred eight or more months following inoculation. This last type, for which the spring peak of malaria was the result of infections in the previous year, was known in northern Europe, for instance, in the Netherlands, Germany, Scandinavia and Central Russia [111]. In historical England, latent primary infections (and relapses) could also cause spring malaria and even deaths [59,112]. It seemed that generally the proportion of infections with a short incubation period (12–17 days [113]) declined steadily with increasing latitude (and shorter summer mosquito breeding seasons) [111]. In Provence which is a southern area, *P. vivax* with short incubation period was implicated; but, it is not possible to completely exclude the presence of strain(s) with long incubation periods. However, on the basis of the relative low number of mentions of spring fevers, if these strains were present, they would at least be a minority during non-epidemic periods. In addition, mentions of spring fevers most likely reflected malaria relapses than latent primary attacks. Moreover, in Provence, the relatively frequent mentions of quartan fevers during winter also suggest that during this season, tertian fever relapses were rare [25].

Although the worldwide spread of malaria is dependent on the presence of vectors, it has always been and still is contingent on human beings (and human activities), and the flux of infected people from endemic areas can play a role in the spread of malaria [114]. The proportion of imported malaria cases due to immigrants in Europe has increased during the last decades, with higher rates associated with settled immigrants who travel to visit relatives in their country of origin [115]. No doubt in the past, foreigners and the French returning from endemic areas also played a role in the spread of *Plasmodium*. For example, even if western Provence was not concerned, during the malaria epidemics in French rice fields in 1741, new strains of *P. vivax* were likely introduced by workers from Italian rice paddies [116]. Even if mass population flux did not occur in Provence, returns or arrivals of people from endemic areas must be mentioned as there are several proofs, both experimentally and in the field, of the healthy human carrier as a source of anopheline infection [29]. During great trade fairs held each year, and more often during the season most favourable to the spread of malaria, there was a flow of people from outside the studied area. Moreover, during the Republican and Imperial wars (1792–1815), the traffic and return of potentially infected soldiers from highly endemic countries such as Italy, Spain, Flanders, or Egypt [117-119] could have permitted the introduction of new strains of *Plasmodium*, but this point did not result in epidemics in Provence. However, this trend occurred during the period when great malaria epidemics had ceased (i.e., after 1776). Similarly, no increased number of autochthonous malaria cases was observed in Provence, although for example, in 1830s and 1840s, 16 000 soldiers who were mainly suffering from malaria were transferred from Algeria [120,121].

These data suggest that during the period of the decline of malaria in Provence, the conditions were not conductive to the spread of alien strains of *Plasmodium*. If quinine use proved to be effective before the 1820s, a low number of vectors and/or a partial resistance could be the most likely explanations. However, the hypothesis implicating a decreased number of *Anopheles* vectors in the decline of malaria in Provence was refuted for eastern Provence, as even in the 19^th^ century, short-distance travels of infected individuals played a role in the spread of malaria that had not failed to be observed by locals. Due to intermittent fever pressure, there was a continuous lack of manpower for farm work in Arles, and residents of neighboring regions were needed [85], with those from mountainous areas particularly sensitive to malaria. Thus, it is not surprising that except during some epidemics and in highly endemic areas, fevers most often preferentially affected day labourers [85,108] who, depending on the year, constituted most of the patients who presented for treatment with intermittent fevers in a Marseille hospital [122].

### Data concerning the intermittent fevers epidemics in Provence from 1745 to 1850

The concept of epidemic implies the temporary increase in malaria incidence with, in most cases, a return to normality. However, in a highly endemic area, an epidemic is generally characterized by an increase of both morbidity and mortality rates and by severe clinical signs that frequently corresponded to mentions of pernicious fevers in Provence. Moreover, epidemics differ not only in their causality but also in their form of presentation, evolution, incidence by age groups, severity and socioeconomic impact [123]. Yet for almost all of the malarial epidemics in western Provence, these characteristics are not well known or are even totally unknown, such as the morbidity and mortality rates by gender or among children. This virtual impossibility to establish a typology of malaria epidemics makes it difficult to determine the factors that could be involved in their emergence. In other words, in this article, various epidemics of intermittent fevers that had different characteristics in terms of morbidity, mortality, clinical signs, duration, and affected populations have been grouped under the same term of malarial epidemics. For example, in Figure 2, the epidemics from 1751 to 1780 were of different types than those from 1781 to 1850, but although it is impossible to determine precise typologies for the epidemics in Provence, some generalities can be made.

The analysis of records evidenced different durations for the epidemics of intermittent fevers. Some of the epidemics were short-lived and only mentioned during a single year by author witnesses, whereas during severe episodes, epidemics generally persisted for several years (from two to five years or more). Examples of an epidemic of two years and one of seven years are detailed below.

A detailed report on the malarial epidemic of 1765–1766 that ravaged the village of Aramon was provided by a local physician [32]. This village is located in the south of Avignon on the right bank of the Rhone River (see Figure 1) and at an altitude around 10–20 m. The physician began his description by emphasizing the deleterious location of the city in the middle of the marshes and next to the Rhone, which had overflowed during the previous years [61]. During the first year (1765), the epidemic of intermittent fevers, which presented with ordinary symptoms of malaria, began in April and ended in September. Fatal cases were rare; patients were easily cured by local remedies, suggesting a number of cases of self-healing. During the following year, the epidemic affected not only Aramon but also various localities of Provence including Arles [31,32]. At Aramon, the epidemic began in February, progressed in May-June and plateaued in July, August and September, thereafter declining [32]. Starting from May, the patients had continuous fevers that then became intermittent; red urine and body aches were also mentioned. During the summer, the number of patients increased and more severe symptoms associated with a higher mortality rate were noted. Fevers were of double tertian type (e.g., malaria episodes in which the paroxysms occur daily are usually named double tertian malaria, representing an infection by two distinct groups of *P. vivax* parasites) or more (i.e., triple tertian, etc.), and patients had convulsions and delirium. Moreover, many elderly died. During October to December, the return of intermittent fevers was regularly observed; however, when they were associated with dysentery, they were most often fatal. During the two epidemic years, 2,000 people suffered from fevers out of the 3,000 living in the municipality, and 136 people died. The deaths mainly occurred during the second year, and 77% of the deaths occurred from July to December. The morbidity rate was, therefore, 66%, with a mortality/morbidity ratio of approximately 5% [32,108]. However, this ratio was underestimated because clinical signs of malaria may not be specific (e.g., fevers without intermittent fits). Based only on fevers exhibiting well characterized fits, the great majority of them were tertian (simple, double, etc.), suggesting that the primary infectious agent was most likely *P. vivax,* but a significant involvement of *P. malariae* must be underlined as during November 1766. Concerning meteorological data, a deficit of precipitation compared to the 85-year average was observed from spring 1765 to autumn 1766 with the exception of autumn 1765; moreover, the summer and winter of 1764 were particularly wet. At Marseille, the mean temperatures from June to September were 20.7°C and 21.1°C in 1765 and 1766, respectively.

Several authors mentioned that in Provence, malarial epidemics lasted from five to seven years [124-126], but accurate data concerning the year-by-year epidemic are quite rare. However, a very detailed description of an epidemic of intermittent fevers lasting seven years (1772–1778) has been made by Got [37] in her medical doctoral thesis based on reports of the Commission on Epidemics (created in 1766) and archives. This epidemic principally affected a small town (Villeneuve-lez-Avignon) located along the Rhone River across from Avignon, and the episode summarized below could be a textbook case study. Due to widespread flooding in previous years, the course of the Rhone River changed, leaving dead arms that turned into lagoons of stagnant water. Later, these pools were implicated in the epidemic a posteriori. The first two years of the epidemic (1772–1773) were also mentioned retrospectively when the latter greatly increased in intensity. In these two years, people who normally were only slightly susceptible to malaria infections were affected; however, fevers, at least during the first year, were relatively benign (quotidian or tertian fevers). The symptoms became more severe over time, and the epidemic peak was reached in 1776. According to physicians from the Faculty of Montpellier sent to Villeneuve-lez-Avignon in 1776, patients suffered from fevers that could be divided into four classes [127]. In the first class, patients had benign intermittent fevers (generally tertian). In the second class, people had intermittent fevers with a comatose state, dysentery and symptoms similar to those of cholera morbus, and they were termed “pernicious intermittent”. Moreover, intermittent fevers turned into continuous fevers [37]. The prognosis of these fevers was very poor, as patients can die during the fits. In the two other classes, patients had continuous fevers, but could also be ascribed to malaria. During the year 1776, two thirds of the population was affected and 5% died. All areas of the city were affected; however, inhabitants living near marshes were most affected both in terms of morbidity (75-100% versus 25-40% for the most remote districts) and mortality (approximately 10% versus 3%). Mortality was null in the two monasteries and in a fort suggesting elevation and surrounding walls could have play a protective role. Moreover, the highest mortality rates observed in areas where many people were poor, suggesting a relationship with undernourishment. Furthermore, patients living near the Rhone River suffered from numerous relapses with short periods of remission, suggesting multiple malaria infections that could be correlated to the number of infected mosquitoes. Early in the year 1777 (until April), the tail of the epidemic was observed with patients with pernicious symptoms, whereas from early summer, the sick largely constituted those who had escaped the disease the year prior, and they were usually affected by benign fevers [25]. This pattern suggests on the one hand the presence of at least different strains of *Plasmodium* and, on the other hand, that some people had not developed acquired immunity against the benign strain(s). In 1778, most patients consisted of convalescents having great difficulty to get healthy. During the years 1772–1778, epidemics of intermittent fevers associated with high summer-autumn mortality affected many other cities of the areas of Avignon, Arles and Berre), but these epidemics were shorter lived and generally less severe [25,37,127]. According to Got [37], the mortality rate and the clinical neurological signs of the epidemic of Villeneuve-lez-Avignon could suggest the implication of *P. falciparum*. From 1772 to 1778, the average temperature recorded in Marseille during the months of July and August ranged from 21°C (1773) to 23.1°C (1778) and was 22.2°C in 1776. According to the data from Marseille, rainfall from 1772 to 1777 was generally lower than that from 1761 to 1827, but precipitation was much higher than the seasonal average in summer 1772 and 1774, in spring 1776 and in winter 1772 and 1776. Low rainfall occurred in all seasons in 1777, the year of the end of the epidemic infection. In summary, generally in Provence, the various stages of a long epidemic (≥5 years) were as follows: during the first years, there is a slow progression in epidemic intensity, and the early stages of an epidemic may often pass unnoticed. Next, an epidemic peak was observed during one or, more rarely, two years, ending with an epidemic tail during the last year. It may be necessary to add a further year during which convalescents were observed to have difficulty returning to health.

Analysis of historical documents brings out some generalities regarding malaria epidemics in Provence. In this region, moderate to major epidemics occurred relatively frequently but at irregular intervals. Spring intermittent fevers were usually both rare and mild. However, during epidemics, especially those raging for several years, spring fevers appeared earlier and ended later, even continuing until the appearance of summer fevers. However, during the first year, spring fevers might remain benign, but this situation was not the case during the following years. If winter infections and those with long incubations are excluded or rare, there must be relapses in the previous year. In highly endemic areas, relapses were usually benign, but this situation was not the case during epidemics. In the summer of the first year of an epidemic, new symptoms began to be observed related to high mortality such as among pregnant women and the elderly [31]. During the year corresponding to the epidemic peak, severe clinical signs were mentioned, frequently including neurological syndromes. Moreover, during great epidemic episodes, the disease could also rage in nearby towns. Generally, witnesses assumed that these epidemics were linked; however, as during the great epidemic period that raged in several parts of Provence in the 1770s, the analyses of records do not permit to conclude whether this pattern reflects disease diffusion or spontaneous emergence [31]. During these episodes, in some years, epidemics were triggered, whereas the weather may not be very favourable in terms of temperature and precipitation. This point constitutes one of the difficulties in determining the most favourable weather conditions for the emergence of an epidemic. Moreover, the impact (morbidity and mortality) and the symptoms observed could constitute substantially different epidemics according to the localities. However, when epidemics occurred in other cities at least one year after the beginning of the epidemic in the neighboring town, the traffic of a large number of convalescents and relapsing persons, associated with a necessary significant mosquito burden, could explain some of these secondary epidemics. During longer epidemics, a relatively slow but continuous progression was observed during the first years, which could be a feature, at least in Provence, of highly endemic areas involving a large number of pre-immunized people. In non-endemic areas, the epidemics are triggered quickly, as observed after the development of a rice field in Thiers (Central France) in 1741 [128]. This hypothesis would imply that pre-immunized people could become sensitive to some of the variants that appear during the epidemic suggesting that *Plasmodium* variants could appear in the course of the epidemic; some strains could be more virulent or have greater epidemic potential, implying also that locals had not developed cross pre-immunization against these variants, which would have antigenic changes.

In western Provence, before 1776, major malaria epidemics affected several cities simultaneously with clinical signs considered as pernicious and with high morbidity and mortality rates. After this breakpoint, epidemics become rarer and more localized; in addition, a change in the nature of epidemic intermittent fevers was observed, with a trend towards more benign and shorter episodes, as outlined in the writings of this period, although some episodes were still very deadly, such as those affecting foreigners. Moreover, most of the epidemics were due to anthropic causes as the digging of the canal from Arles to Bouc. This period of transition of the late 18^th^ century, although not always precisely dated, had already been perceived by contemporary authors and was confirmed by others later [18,46,85,105,129,130]. However, if the epidemic breakpoint can be dated with some accuracy, such is not the case for the endemicity, although it may be considered that from about the same time it began to decline.

In high endemic areas, due to the great level of premunition in the population, morbidity and mortality occurs mainly in early childhood and in pregnant women and epidemics generally do not occur [75] and epidemics are rare, indeed, most epidemics occur in areas of low endemicity or as a result of the mixing of infected and susceptible individuals and thus originate from a rather larger reservoir in a population not fully susceptible [123]. However, in highly endemic areas of Provence a very high frequency of severe epidemics has been observed. Moreover, no mention of significant flux of naive people has been found in historical documents. In addition, given the rate of endemicity that persisted in the studied areas, there was no important change in the level of acquisition of natural immunity. Epidemics of the early 19^th^ century during the digging of the canal from Arles to Bouc are particularly informative because even during the period where the pressure exerted by malaria had declined, almost the entire indigenous population was generally unaffected. However, on one hand, even if the premunition rate should be high among a large proportion of the adult population, urban/rural areas and poor/wealthy variability should be evident. On the other hand, the putative implications of nonimmune populations in otherwise malaria-free locations adjacent to endemic regions and in elevated villages within these same regions could have played a deleterious role, but their impact was most likely minimal. Epidemics could also be the result of the invasion by an highly efficient vector that finds a permanently suitable environment [123], but on one hand, if malaria was already stable this invasion should not have consequences, and on the other hand, in Provence, even in the first half of the 19^th^ century, seasonal workers were frequently involved in the spread of malaria, suggesting that the number of efficient vectors was still quite high.

### Analyses and consequences of human activities

#### Agricultural practices

Agricultural practices can induce variation in the number of potential anopheline breeding sites and thus modify the abundance of anopheline populations, with possible consequences for malaria. So, various data concerning agriculture are discussed below.

In the 1920s, Roubaud stated that it was the relationship between anophelines and cattle that regulated the incidence of human malaria; human beings could be protected from exposure to mosquitoes by stabling domestic animals close to anopheline breeding places [131]. Likewise, in Britain, 9% of the drop-off in malaria can be attributed to an increased cattle population [132]. However, assuming that this hypothesis was correct, during the analysed period in western Provence, there was no real change in the animal husbandry (such as stabling the cattle or keeping them outdoors at night) and in the ratio of cattle to sheep [41].

Olive oil was a major source of food and of financial input in Provence [133]. During episodes of high mortality of olive trees, it is stated in the archives that the inhabitants were reduced to their greatest misery [25]. However, no correlation has been found between the years with major freeze olive-trees damage ([42-44] and Additional file 1) and those with malarial epidemics in at least one area (Chi-squared test of independence, p-value = 1).

Both in the 18^th^ and 19^th^ centuries, several authors had made a connection between the retting of hemp (*Cannabis sativa* L.) and flax (*Linum usitatissimum* L.) and the prevalence of malaria [1]. According to ancient authors, intermittent fevers could be the product of emanations from putrefying vegetable substances. But later, the larvae of anopheles were demonstrated to be unable to live in the water resulting from this process [134,135]. However, the network of canals and ditches constructed to bring water for the retting process provided potential breeding sites for mosquitoes. Nevertheless, in Provence, the putative impact of this type of cultivation on malaria would be relatively low, as it concerned only small areas [37,136,137].

Rice cultivation has been associated with increased transmission of intermittent fevers in many areas, and populations often resisted this cultivation in the name of public health. This type of crop was even repeatedly prohibited in several regions of the world, including in Provence [116]. In the 19^th^ century, timid attempts at rice cultivation took place near Tarascon in 1829 and in Camargue from 1844, but large-scale cultivation in the latter region began in 1848 [41,45]. So, this shows that rice fields cannot be involved in the malaria fever level during the studied period, even in the last years of the first half of the 19^th^ century.

In the 17-18^th^ centuries, physicians and historians underlined a causative correlation between the invasion of locusts and epidemic fevers and even a causal link, as intermittent fevers would have been due to the emanations of putrefying bodies of these insects. Indeed, locust plagues appeared often in spring and intermittent fevers in summer; and the insects that finally died generated nauseating odors and could drown in large numbers in ponds, rivers and other water points. However, in the 19^th^ century, some scientists were skeptical of this theory and demonstrated that the observed temporal sequence did not demonstrate causality [138,139]. Nevertheless, from a minimalist point of view, these two events could have the same underlying cause, and putative correlations between locust invasions, epidemic outbreaks and weather conditions (temperature and precipitation) were investigated. However, in western Provence, no correlations were found between locust plagues [140] and the meteorological conditions supposed to be favourable for the spread of malaria, which is not surprising because in low Provence the periodicity of mass outbreaks of locusts is connected with xerothermic climatic episodes (droughts with high summer temperatures) [39].

#### Socio-economic data

Many malaria epidemics follow or coincide with periods of economic crisis, war, civil disturbances or human migrations, affecting impoverished or displaced populations that are not just physically weak [116,123,141]. Yet during the analysed period, except during the first part of the revolutionary time (1789–1794), the period from 1745 to 1850 was a relatively peaceful time in western Provence. Moreover, no great malaria epidemics were mentioned during the troubled period that was the French Revolution; however, this period was situated after the breakpoint. The impact of epidemics not only depends on the increase in specific morbidity but also on the general health of the affected population. Many epidemics due to malaria or other diseases coincide with periods of famine, affecting already undermined populations unable to obtain appropriate medication [123]. Additionally, malaria is frequently referred to as a disease of the poor or, at least in part, a “poverty disease”; hence, the combination of under-feeding with conditions favouring infection is irresistible [142]. This theory is also supported by the observations in the 20^th^ century that malaria prevalence and mortality were generally lower among the wealthy [143]; however, there is no evidence to indicate that this link is more than a coincidence [144]. Indeed, recent literature on the relationship between malaria and nutrition is controversial; however, nutritional deficiencies are frequent in malaria-endemic areas, and it seems that vitamins could play an important role in the proliferation of malaria parasites [145,146]. However, even if malnutrition and malaria epidemics are not considered to be directly linked, their inter-relationship is generally accepted as being synergistic, with one promoting the other. For those who contract this disease, the prospects of recovery are often diminished by their poor nutritional status. Malaria has long been recognized as particularly lethal amongst people weakened by malnutrition. Indeed, the Tuscans have an old saying, “*la malaria si cura nella pentola*” (the cure for malaria lies in the cooking pot), which is frequently cited in the scientific literature since the mid-19^th^ century [147] is but most likely much older; this saying would also have existed in French [148,149]. In Provence during the studied period, there were no true famines but rather scarcity episodes, which were relatively rare, i.e., during the years 1747–1749, 1771, 1773 and 1797 [150]. A correlation between malnutrition and epidemics was only found for 1773, and even if it is during the last great epidemic period, this is inconclusive. However, interestingly, during this period (1772–1777), according to contemporary physicians, the poor have generally paid the heaviest price in terms of mortality [37], even if in some cases, all the social classes were infected in an equivalent manner [25]. In 1810, a physician near Berre noted that repeated bouts of malaria may expose individuals to chronic malnutrition [20]. Therefore, as people lose productive time through illness; economical consequences should be taken into account in studies of malarial endemicity in Provence. In the underprivileged classes, when the fathers of families were sick or died, even children started working very young, which had repercussions on the survival of the family in terms of food and health [25]. Moreover, the physician was called only when the poor patients were close to death [151], and poor patients could not buy remedies. In addition, because of the financial instability at the family level, a large number of people who were infected by malaria might succumb during the winter [37]. During each malaria attack, a person loses the equivalent of 3 days of food for an adult [152]; therefore, for those with a poor nutritional status, the prospects of recovery are often diminished after malarial infection. Furthermore, outside of the major epidemic periods, local low malaria epidemic episodes could generate food shortages that affected the persistency of the disease as farmers were no longer able to harvest. For example, in the second half of the 18^th^ century, a physician of Aramon mentioned that marsh fevers were responsible for the scarcity of grain and hay [108].

Albeit unquantifiable, vitamin deficiencies, principally scurvy, were frequently mentioned in French marshy malarial areas [1]. In the studied area, physicians mentioned that at Fos, the population was miserable, poorly fed and often suffered from scurvy [153]; similarly, the people of Marignane also suffered from scurvy [154,155]. Even if not been proven clinically, vitamin C (ascorbic acid) may act as a pro-oxidizing compound on advanced plasmodia development stages, and there is no doubt that the Mediterranean diet, usually rich in ascorbic acid-containing food, may be responsible for some protection against malaria; this cultural basis contributed to reduce disease risk, as extensively reported by the specialized literature [156]. However, in France, scurvy could be facilitated by the fact that the consumption of fruits, even occasionally, was often discouraged because they were often blamed for various gastric disorders, including dysentery. For example, in a village in Provence, an excessive consumption of fruits was incriminated in the first death due to cholera during the epidemic of 1835 [157]. As gastric symptoms can also be observed in malaria, consumption of fruits could also be considered as the main cause of intermittent fevers [128,158,159]. Even in Africa, consumption of fruits is considered one of the causes of malaria [160]. Anecdotally, at Berre, peaches were blessed on the day of the Saint Caesarius of Arles, who was most likely the first known case of autochthonous malaria in Provence and was also supposed to cure intermittent fevers [161]; the belief that these fruits can cure fevers was were very common in this village [46]. More generally, fruits could be distributed during patronal festivities [46]. Without making any correlation, a physician of the Berre area where scurvy was very frequent noted that in the year 1809 there were no fruit crops and there was a greater prevalence of malaria, unlike previous years that were almost malaria-free and during which fruit crops were abundant [20].

#### Civil engineering works

Man-made structures involving great movements of land can provide suitable larval habitats for mosquitoes and have often been accompanied by malaria epidemics among workers and neighboring populations, even in areas with very low levels of endemicity (references in [33,162]). The slightest road construction requires materials taken from holes that can remain alongside and provide suitable sites for anophelines. In the studied areas, during the second half of the 18^th^ century, local people and physicians frequently considered civil works as responsible for the onset of epidemics, such as the digging of canals at Tarascon in 1766 and in the east of Avignon (Morières) from 1778 to 1781 or the construction of a breakwater and/or roadway at Villeneuve-lez-Avignon in 1774–1776 [25,37]. However, epidemics were so frequent during this period that it is very difficult to determine whether these works could have had major sanitary implications. Moreover, excavation works were performed without mention of epidemics or even of an increasing number of sick patients. The probable increase in the number of sick during these civil works was lost in the background of the high level of endemic malaria. Furthermore, even if this theory is an argument without data, the use of indigenous manpower could reduce the deleterious health impact. For this reason, as described below it is focused on the two great civil engineering works conducted during the first half of the 19^th^ century.

##### Construction of railway lines

In the 19^th^ century, malaria was noted to spread along the new railway lines constructed in Italy [33,90,163,164]. Similarly, in France, the construction of railways could be a cause of the spread of malarial infection even in relatively healthy areas [165]. In Provence, during the analysed period, the main railroad line was constructed from 1843 to 1848 from Marseille to Avignon. The railways passed through the three studied areas, but only the edge of the area of Berre had a higher rate of endemic malaria. However, an analysis of historical documents does not reveal any significant increase in the rate of malaria infection, with the exception of the following mention: according to a contemporary eyewitness, since the establishment of the railway, the plain on the south of the Durance River (at the East of Avignon) that it crosses was *cruelly ravaged* by intermittent fevers, even obliging the farm population to suspend their works [166]. Yet the mention of “*cruelly ravaged*” must be set in context and could be explained by the fact that malaria had become relatively rare. Unlike Italy, the sanitary impact of the construction of railways in Provence has been relatively limited. Some attempts of explanation can be advanced; the railway line crossed through regions of Avignon and Arles with malarial endemic rates that had become relatively low at the middle of the 19^th^ century. Even if severe epidemics are often found in areas of low endemicity when conditions are favourable, this case did not apply here. The railway passed only at the edge of Berre, a region where malaria was still very present although declining. Moreover, an analysis of clinical signs when mentioned exclude the involvement of *P. falciparum,* and quinine was given to workers and relatively quickly cured the patients.

##### Digging of canals

The most deleterious malarial impact during engineering works was frequently observed during the digging of canals [167]. In western Provence, several canals of various importance were dug during the period analysed [15,25]. An analysis of texts concerning this region demonstrates that although the digging of several canals was linked to epidemics, only one had a substantial and unambiguous (because after the breakpoint period) impact in terms of malaria epidemics: the canal from Arles to Bouc [46,47]. This canal connects the Rhone River near Arles to Bouc on the Mediterranean Sea, and it is 47 km long with one lock (Figure 1). This canal should allow ships to avoid the unfavourable navigation conditions in the lower part of the Rhone.

The study of the epidemic outbreaks around this canal is interesting for several reasons: 1/ the significant health impact on canal workers but also, in some cases, on autochthonous populations in the neighboring areas; 2/ it occurred during the period (first half of the 19^th^ century) when the pressure exerted by endemic malaria declined; 3/ the area (highly endemic area of the Etang of Berre); and 4/ the characteristics and symptoms of the disease. The engineering works started in 1802, were halted in 1813, and resumed in 1823 until 1842, although the canal began to be open to navigation from 1834 [47]. Difficulties during the digging were numerous: the canal traversed swampy malaria-endemic areas over the major part of its route; it was necessary to dig through particularly hard sedimentary rock at the southern end of the canal; and there were many uncertainties and political and financial fluctuations due to the Napoleonic Wars. In addition, the large number of illnesses and deaths created manpower shortages and thereby affected the progress of the works [168].

As most frequently observed during major earthworks, there is the creation of new mosquito breeding sites and the influx of workers from outside the region, most of them “immunologically naive” and other potential carriers of particular strains of *Plasmodium*. So, an increasing number of patients were observed in the region with, in some cases, a clear transition from endemic to epidemic disease as evidenced by the archives [69,169,170].

The number of workers varied over the years. Some years, up to 2,000 labourers worked on the canal [168,171], but this number was significantly lower in the early years. There were voluntary workers but also from 1806 until 1813 many military deserters and refractory conscripts of the Napoleonic army, the latter requiring surveillance personnel (gendarmery) [20]. In addition, in the same area, Swiss and French soldiers and customs officers were assigned to the coastguard, and some of them came with their families. All of these people, whether free or prisoners, usually lived in the midst of malarious areas and were mostly poorly clothed, fed and housed. The last mention suggests that workers could be bitten while they slept in their makeshift dwelling; but during the summer-autumn period, the autochthonous population should also been bitten at a high frequency. Moreover, the worst off were the prisoners who arrived very meager at the hospital. Although precise data are lacking, a malarial morbidity rate of 100% was mentioned for some years, such as 1811, with rates of mortality among workers above 20% [25,46,68].

Before the digging of this canal, the last major epidemic around the Etang of Berre took place during the summer and autumn of 1789 with a 100% morbidity rate and 36% mortality rate among the autochthonous population in some villages; the epidemic constitution was quite complex. However, pernicious intermittent fevers were noted to kill many people [20]. From 1801 to 1803, after floods, a significant number of deaths due to intermittent fevers were mentioned in Arles but not around the Etang of Berre in 1801–1802 [25,61].

During the period of digging, the first epidemic was mentioned during the second year (1803); at Fos, during the summer alone, more than 450 cases of intermittent fevers were observed, affecting nearly two-thirds of the inhabitants of the commune [172]. The number of deaths is not mentioned, which may suggest that this figure was very low. For this period, the registers with monthly temperatures have been lost, but annual average temperatures are always available, which is slightly lower than the mean calculated for the period 1745–1850 (13.9°C/14.1°C). Considerable excess rainfall was observed in October and November of 1802 and in January 1803, but the remainder of 1803 was particularly dry [172]. The epidemic episode of 1803 shows that despite the endemic situation of this area, and contrary to what was generally observed in the following years, the autochthonous population did not seem to present any resistance in terms of infection against the *Plasmodium* strain(s) present at that time. The role of the digging of the canal in this episode cannot be verified, but the episode could be due to the importation of new *Plasmodium* strain(s) or to the emergence of virulent variants.

During all the years during which the canal was dug many workers fell ill. From the start of the digging of the canal (1802) until the temporary halt after 1813, the works were mainly in the southern part of the canal; therefore, persons seriously ill were generally sent to hospitals of the region of Berre, principally at Martigues. Interestingly, from 1805 to 1809, a physician of the hospital of Martigues who specialized in the treatment of fevers provided precise data concerning the epidemic condition [20]. According to him, there appeared to be a correlation between rainfall and flooding and the number of cases of fevers. The fall and winter of 1804 were very rainy whereas the year 1805 was very dry, except in January. In 1804, malaria had little impact, whereas from spring 1805 to March 1806, a large number of simple intermittent fevers were observed except during the summer of 1805 when many fevers were pernicious (i.e., with severe complications) [20]. Those affected were men who worked on the canal; of note, there was a cinchona shortage during this period due to the Napoleonic Wars [20]. From spring 1805 to March 1806, a high frequency of intermittent fevers was observed among the autochthonous population living around the Etang of Berre, which may be associated with other diseases, and a relatively high number of abortions were also noted [20]. In 1806, a physician mentioned that in Tarascon (a town north of Arles) pernicious intermittent fevers were much more common than in previous years [25], suggesting that malignant *Plasmodium* strains could be present in this region.

The years 1806, 1807 and 1808 were very dry, whereas in the summer of 1809, small frequent cold rains that delayed crop maturity were recorded. However, during the months of July and August 1809, the average temperature at Marseille was 21.2°C, more than 2°C less than those measured during the previous three years. During the years 1806, 1807 and 1808, only a few fevers were recorded, whereas in 1809 more fevers were recorded in the hospital that over the previous three years [20]. The pernicious fevers were however rare, affected predominantly patients with simple intermittent fevers, although some suffered many relapses and long convalescences. There were few patients among the rural or urban autochthonous population of the surrounding communes; indeed, these fevers affected almost only the “foreigners” newly arrived, i.e., both free and prisoner workers employed in digging the canal and the soldiers (Swiss or French). In addition, people who came from outside this area (free or prisoners) were affected in a similar way. Until 1809–1810, even among the most disadvantaged people (the deserters), the malaria mortality rate was very low. The hospital doctor also noted that it was important to stress that the canal workers who had most suffered from intermittent fevers in previous years were protected in 1809 [20]. He also notes that in five years, none of the prisoners who constituted the most miserable part of the hospital population appeared to transmit their infection to the other patients. This last point, associated with many mentions of intermittent fevers, strongly suggest the involvement of malaria. When specific data were mentioned in the records, the distribution of various types of simple intermittent fevers were as follows: 68%, 11% and 20% for tertian, quartan and quotidian types, respectively. Moreover, quotidian attacks frequently became tertian. During the last three years of the first phase of the canal digging, not only the morbidity rate was very high, but unlike in previous years, many deaths were recorded. The most disastrous year was 1811, during which at Arles and at the hospital of Martigues more than 400 deaths, mainly due to intermittent fevers, were listed among the personnel working on the canal. During the same year, it was indicated that since June, all prisoners and all their military guards without exception were ill [25,68]. There were also many deaths in 1812, and there is no indication that the autochthonous population was greatly affected.

The cessation of the works (from 1814 to 1821) involved not only a cessation of digging, although breeding sites were still present, but also the efflux of many foreign workers from the region, which led to calm in terms of malaria. The majority of patients in 1814 most likely had relapses of infections from the previous year [46]. In 1817, a severe malaria epidemic raged in the Camargue with a morbidity rate up to 60% and mortality rates from 5 to 12% [18], but no increase in the number of sick around the Etang of Berre was reported. In 1821, followed the heavy flooding of 1820, a severe epidemic affected the inhabitants of the Berre area [18]. There is no certainty that this epidemic was due to malaria, especially as the inhabitants described this epidemic as a plague [80] and also due to mentions that intermittent fevers were relatively rare at Fos during the period corresponding to the absence of the works [173].

The contemporary witnesses of this period observed that the recovery of the works since 1822 again had epidemiological consequences [46]. However, the year 1822 was very dry without a great epidemiological impact; however, according to these witnesses, malaria returned again in 1823 due to the floods but with less serious consequences in terms of mortality, although fevers could still be pernicious, principally in autumn [46]. In 1823, an epidemic of fevers raged in the city of Arles that, according to a “second hand source”, caused nearly a thousand deaths. In the same year, an epidemic of intermittent fevers had a severe impact on the workers of the canal [25]. From July 1826 to the first months of 1827, there was again an epidemic episode with a morbidity rate higher than 25% among the workers of the canal. This epidemic also very severely affected the autochthonous population of the communes close to the area where the canal opened to the sea [98], where in some cases, two-thirds of the inhabitants, and all of the children, suffered from intermittent fevers [174]. More precisely, at Fos, from July to August 1826, the morbidity of the population suffering from intermittent fevers exceeded 13%, and in some of the surrounding villages, a malarial morbidity rate higher than 50% was reported from 1826 to January 1827 [18,80,175]. However, the quinine, although very expensive, was increasingly used and thus could explain the low mortality. During these great epidemic episodes, due to the high number of sick workers, healthy labourers were reluctant to return to the malarious area, so lack of manpower arose, which led to a slowdown in the progress of the work. In October 1840, almost all of the inhabitants of Fos were affected by intermittent fevers, but there were few deaths and no indications concerning the workers of the canal [176]. According to the witnesses, due to extraordinary flooding in November 1840 and October 1841, intermittent fevers raged strongly along the canal throughout the summer and through part of the fall of 1842 [47]. This epidemic of intermittent fevers was also reported in Arles [25,177,178]. This episode is the first epidemic of malaria after those of cholera (1832, 1835, 1837) but also the last major epidemic in this malarial zone, and although the morbidity rate was high among the autochthonous population living in rural areas, the mortality level was very low.

This brief description of the health status during the digging of a canal highlights the complexity of historical studies concerning malaria. Severe attacks have raged interspersed with periods of calm [80]. Some epidemics have affected foreigners but also very severely the autochthonous population, whereas others concerned only the foreigners. There were also episodes of mild intermittent fevers with high morbidity but a very low mortality rate, whereas at other times, the mortality level due to fevers was very high. Similarly, intermittent fevers qualifying as pernicious per contemporary physicians did not have all the same health impact; however, the low mortality rate observed since 1826 could also be due in large part to the extensive use of quinine and not only to mild *Plasmodium* strains. During the digging of the canal, malarial morbidity was almost always low in some areas of the Etang of Berre; e.g., intermittent fevers had become relatively rare in Marignane [172]. There were also hyperlocalized severe epidemics in some communes that were most likely unrelated to the digging of the canal as at Saint-Mitre in 1828–1832 [80,172,179]. In some epidemic episodes, the high morbidity among the local population and the cases of extreme sensitivity among foreigners during only the first infection could suggest the introduction or the emergence of new strains or variants of *Plasmodium* or that a primary malaria infection could confer relatively quickly a partial immunity upon its host. The complex epidemic constitution observed during some years suggests that the effects of other diseases superimposed upon those due to endemic-epidemic malaria as during the episode of 1821. Moreover, the analysis of the epidemics during the digging of this canal could also suggest indirectly that endemic malaria declined in this region.

Similar to the situation observed during the digging of the canal from Arles to Bouc, at several times, construction on the canal of Panama was halted due to the lack of any healthy workers. By comparison, during the French effort to build the canal of Panama (1881–1888), the total mortality rate among the effective force employed was estimated to 20-28%, whereas the annual morbidity rate was 62.5%. Most of the workers died from disease a short time after arrival. The mortality rate due to tropical diseases with almost exclusively unambiguous; clinical signs was estimated at 4% (1.5% and 2.5% for malaria and yellow fever, respectively) [180]. However, the number of people suffering from malaria was underestimated, as is generally the case, and more of them likely died from malaria than from yellow fever. Moreover, for survivors, there was no “true” immunity to malaria, contrary to yellow fever. However, this point demonstrates that the digging of a canal in an endemic area of western Provence, although carried out during a period when malaria pressure had declined, had more consequences in terms of morbidity and mortality than those observed during similar works in a tropical area; in Provence, the agent of yellow fever was not present and the involvement of tropical *Plasmodium* strains and *P. falciparum* not proven. Furthermore, it must be noted that in Camargue in the 1910s, several deaths were observed during the digging of ports or maintenance of canals [105], showing that in the 20^th^ century, civil works in the last endemic area of Provence always had deleterious consequences. Interestingly, in the other parts of France, most of the major malaria epidemics during the first half of the 19^th^ century apparently had an anthropic origin and could have raged in some cases in much less malarious areas than western Provence [127,165,181-183].

### Characteristics and symptoms of Provencal intermittent fevers and *Plasmodium* species involved

#### Putative implications of *Plasmodium falciparum*

A variety of elements could suggest the plausible implications of *Plasmodium falciparum* in the Provencal epidemics. First, there were pernicious complications associated with high morbidity and mortality, which are more reflective of *P. falciparum* than *P. vivax* involvement. Further, the results obtained from the temperature diagrams (Figure 3) indicate that a potential malaria transmission risk of *P. falciparum* during four months (June to September) did indeed exist in western Provence during most of the years of the studied period. Indeed, for example, from 1745 to 1792, the period for which there are continuous daily data, the two minimal mean temperatures in July and August were 20.4°C and 21°C for a global mean of 22°C. During some years, the conditions were particularly favourable; for example, in 1774, a link was made by witness authors between extreme heat (mean temperature at Marseille in July and August: 22.6°C) and an increase in the number of intermittent fever cases in Arles [184]. It turns out that for nine consecutive days at the beginning of the month of August, the average temperature was above 27°C and the daily mean temperature was always below 26°C. At a mean temperature of 26°C, 8–9 and 11 days are sufficient for full sporogony of *P. vivax* and *P. falciparum,* respectively [74]. However, during this period, over five consecutive days the temperatures measured at 2:00 pm were higher than 33°C, a temperature at which the development of the parasite in *Anopheles* stops [185].

When daily temperatures dip below 18°C, the possibility of *P. falciparum* sporogony is blocked. However, in the 1920-1930s, tens of thousands of (clearly) indoor infections, many caused by *P. falciparum*, occurred as far north as the Arctic seaport of Arkhangelsk (61°30 N), showing that this parasite can overcome the outside temperature constraints [104]. Nevertheless in Provence, if during the hottest period of summer and early fall both indoor and outdoor infectious bites could occur, during the remainder of the year epidemiological data suggest that indoor transmission was rare or quasi null even.

In terms of the persistence of the organism and the high temperature of sporogony, *P. falciparum* is the least resilient *Plasmodium* species, and if this species was implicated in epidemics in Provence, as climatic conditions may be unfavourable, this theory would require repeated reintroductions. However, it is well known that malaria (including those due to *P. falciparum*) can be imported by immigrants and natives returning from endemic areas. Moreover, cases of late occurrence and chronic asymptomatic carriage of *P. falciparum* malaria have been reported [186]. This species was present for a long time in southern Europe (Italian and Iberian Peninsulas and the Balkans) [187,188] including in a neighbouring region (the Italian Peninsula) of Provence at latitudes higher than in Provence [189]. However, as previously shown carriers arriving from foreign countries do not seem to have been involved in epidemic outbreaks.

#### Severe malaria due to *Plasmodium vivax*

It is assumed that elsewhere in the past in Europe, including in Scandinavia and Russia, *P. vivax* was the major malaria pathogen until its official eradication in 1975, judging from the (tertian) intermittency of fever bouts described in humans infected by the parasites, the relatively low severity of illness and its ability to survive cold winters [190]. Indeed, *P. vivax* is flexible and can survive long periods without the vector as dormant hypnozoites in the human liver. It adapts to local conditions and, regionally, displays different relapse patterns that optimize the transmission of the parasite [191]. Long dormancy was generally found in northern areas of the Earth; in the eastern parts of Finland relapses could be statistically detected at least nine years after the primary infection [192]. The risk of *P. vivax* transmission is more than one hundred times higher than that of *P. falciparum* transmission, which is due to infectivity and the differences in the length of the sporogonic cycle [55].

Thus, in Provence during the LIA, based only on the temperature data and the frequency of mentions of well-characterized tertian fevers, possible *P. falciparum* implication in the malaria epidemics in Provence may have been outclassed by *P. vivax*. Moreover, *P. vivax* malaria has long been considered only rarely fatal, but recent studies have revitalized the argument that *P. vivax* may often become pernicious and directly threaten life [193-197]. According to a recent work, in India, the presumption of the benign character of *P. vivax* results in an underestimation of the burden of malaria mortality by at least 9- and as much as 19-fold [198]. In recent retrospective and prospective hospital-based studies (references in [196]) involving 18,141 patients, 13% and 14% were classified as having a serious illness with a diagnosis of *P. falciparum* or *P. vivax*, with 10% and 7% of those ending in death, respectively. Comorbidities are common in malaria-endemic regions, and their importance in contributing to severe and fatal *P. vivax* malaria is likely underestimated [195]. For example, malaria therapy was initially developed to treat neurosyphilis, a debilitating and deadly disease [199]. In 1938, Swellengrebel and de Buck [29] reported that 7.7% of the patients with syphilis who were treated with induced *P. vivax* infections subsequently died; those with other comorbidities were at particularly high risk. Moreover, today, most reports of severe and fatal *P. vivax* malaria come from endemic regions where populations have limited access to healthcare and a high prevalence of comorbidities and where drug-resistant *P. vivax* strains prevail [194]. In a context analogous to Provence before the use of quinine, radical cure was likely extremely rare, and patients could have several relapsing infections and probably co-infections.

*Plasmodium vivax* is now recognized as a cause of severe and fatal malaria; severe *P. vivax* clinical syndromes documented include significant thrombocytopaenia, cerebral malaria (including coma), severe anaemia, and acute renal, hepatic and pulmonary dysfunction (including respiratory distress), and black urine [191,194,195]. During the Provencal epidemic episodes, several severe symptoms and complications generally attributed to *P. falciparum* but that can also occur during the course of *P. vivax* infection are mentioned. This concerns for example, cerebral dysfunction (i.e., altered sensorium, convulsion and coma) [195,200-202], and excretions of orange- or red-colored urine which may be the result of severe hemolytic anemia in sufferers [195,203]. Moreover, it is known that “*repeated attacks of malaria due to any species of the parasites over several to many years severely debilitate body and mind*” [81] and malarial cachexia were frequently mentioned in the area of the Etang of Berre [20,22,105,155,204].

Other historical data also suggest that *P. vivax* can be highly pathogenic [132]. According to Dobson (1980, 1997) [59,86], in estuarine swamps in England, *P. vivax* malaria could also be a great debilitator for various reasons: 1/ inhabitants suffering from malaria would be less able to resist attacks of other infectious diseases, such as smallpox, typhoid, tuberculosis and influenza (*P. vivax* disease severity*,* like that of *P. falciparum,* would be highly context dependent [83,193,205,206]); 2/ many diseases would have been complicated by an attack, primary or relapse, of malaria, whereas the course of malaria itself could have been severely influenced by the presence of other infections; and 3/ repeated attacks of malaria in an area of endemic *P. vivax* were likely to result in a chronic state of ill health and lead to early death. According to Dobson [59,86], in the British marshlands and fens from the 15^th^ to the 19^th^ century, mortality directly associated with untreated *P. vivax* malaria could reach 5%. In Holland at the end of the 19^th^ century, equally high death rates were reported from intermittent fevers [29], whereas in Finland, according to historical reports by the district physicians, the mortality during malaria epidemics usually varied between 0.85 and 3% [48,101].

Even if precise and pertinent data are extremely rare and do not concern the studied period, a very short incubation period has been mentioned in records, which is a criterion that the infectious agent could be *P. falciparum*. For example, in Camargue, in the 1910s, the shorter incubation period – the time from a bite from an infected mosquito (sporozoite inoculation) to symptom development – were 7–8 days [105]. *Plasmodium falciparum* tends to have a shorter incubation period (6 days, even if the median is generally approximately 11 days). However, the frequency of tertian fevers, the rarity of pernicious cases and especially the detection of parasites in the blood during this period and later [105] strongly suggest that malaria was principally due to *P. vivax,* especially as the minimum incubation period for this species is 7 days, whereas the median values varied according to the strains [110].

Even if it seems likely that in Provence, *P. vivax* was the prevalent species and was also responsible for severe and lethal illnesses, puzzling questions remain. Indeed, how to explain that in an area with very high malarial endemic level, intermittent fevers were usually mild except during epidemics, however not in all? In addition, at the beginning of the epidemics that raged during several years, only mild symptoms might be observed, whereas severe clinical signs appeared later. Additionally, how is it that after the breakpoint period severe epidemics raged much less frequently? Some explanatory hypotheses may propose although speculative: temporary introduction of new parasite strains and/or changes in the virulence of the *Plasmodium* strain, as discussed below and/or changes at the acquired immunity level. Changes in the strains of *P. vivax* leading to less virulent or less lethal strains has been suggested in Netherlands [29], in England [83,86,103] and in French Alpine Valleys [207]. However, in historic Finland, a change in the virulence of the *Plasmodium* should not be assumed; according to the authors, the number of deaths from malaria increased when the number of malaria cases increased [101].

### Data concerning malaria vectors

#### Anophelines in Provence

Several past data might help to evaluate retrospectively the status of anopheline populations in Provence during the 18-19^th^ centuries, but extrapolations are uncertain for several reasons [51]. First, in Provence, the first information about *Anopheles* collections was obtained more than a century after the last great malarial epidemics. Second, the literature on malaria vectors, especially in southern Europe, must be evaluated with caution; for example, in the past, the taxonomic relationships of species complexes were not understood [208,209]*.* Third, great faunistic changes may occur over time, such as those observed within this genus during the 20^th^ century.

The more recent studies reveal that nine anopheline species are actually found in western Provence (*Anopheles algeriensis*, *Anopheles claviger s.s.*, *Anopheles hyrcanus*, *Anopheles petragnani*, *Anopheles plumbeus* and those belonging to the *Anopheles maculipennis* complex: *A. atroparvus*, *A. maculipennis s.s., Anopheles melanoon* and *A. messeae*) [51,210]. Although there may be discrepancies between authors, from an epidemiological point of view, only *A. atroparvus,* due to its abundance and its relative anthropophily, may be considered as a dominant vector species of human malaria in past Provence. The species *A. algeriensis*, *A. claviger*, *A. maculipennis* s.s., *A. melanoon, A. plumbeus, A. messeae,* and *A. hyrcanus* could have been implicated as possible secondary or local vectors across their distribution ranges, whereas *A. petragnani,* which has been reported several times in south-east France, apparently plays no role in the transmission of malaria [211-213]. Various data that may be useful in the search for potential vectors in Provence in the past are provided below.

*Anopheles atroparvus* was present in great numbers in several environmental patterns of southeast France from the coast to the subalpine level by the 1940s and 1950s [63,214]. Moreover, in Camargue during the malarial epidemic episode of 1943, *A. atroparvus* was by far the most abundant species of its genus [63]. However, in Provence, a near disappearance of the *A. atroparvus* populations since the 1950s was observed; this species was rarely found during a 4-year survey in the 1970s [215], and recent studies in Camargue confirm this trend [52,53]. The data in Camargue between the two World Wars, which most likely could be extrapolated to a large part of Provence, revealed that *Anopheles* (presumably mainly those belonging to the *A. maculipennis* complex where *A. atroparvus* was predominant) were found in stables, piggeries and poultry as well as in urban areas, but in relatively low numbers in the houses [216,217]. The life span of an adult *A. atroparvus* ranges from 6 weeks to 6 months. Thus adult females during the cool season take refuge in a relatively warm locality (stables or houses) and can infect humans during their periodic blood meal, but these meals do not result in egg production (i.e., gonotrophic disassociation) [211,213]. However, as already mentioned, this type of transmission which has been reported in northern European countries [101] and suspected in the French Alpine valleys [218] does not seem to have played a role in Provence.

In historic western Provence, the *A. hyrcanus* abundance was relatively low [219,220]. Due to rice cultivation, this species is now very abundant in Camargue and seems to have replaced the species *A. atroparvus*[52]. Currently, *A. hyrcanus* seems to be the only vector likely to play a role in potential malaria transmission in the Camargue as it is abundant and anthropophilic [52].

*Anopheles claviger* was spread all over the French Mediterranean coasts in the 1950s [214]. Its epidemiological importance is considered to be not significant due to its small populations [211]; however, this species, reported as zoophilic, will readily feed on man and has been shown to transmit malaria in Eastern Mediterranean towns, where it is an urban mosquito breeding in water stored in reservoirs [209]. Similarly, *A. plumbeus* is an opportunistic species which can breed in urban environments, being able to complete its larval development in small tanks; moreover, infectivity tests have revealed its ability to transmit tropical *P. falciparum*[221]. This species was relatively abundant in many areas of Provence in the middle of the 20^th^ century [214]. Thus, *A. claviger* and *A. plumbeus,* which could be abundant in some places particularly those close to humans [53], could have played a role in past major urban epidemics.

Although the vectorial capacity of *A. algeriensis* is relatively high [211], due to its exophily and relatively low abundance in villages in open fields according to surveys in Provence of the 20^th^ century [53,214], its role might have been negligible.

*Anopheles melanoon* was relatively abundant in coastal areas of Provence according to data from the 1950s and 1970s [214,215], and its abundance has not diminished, at least in the Camargue from the 1950s [53]; however, in this area, *A. melanoon,* which is competent to transmit malaria parasites, is almost exclusively attracted by large mammal hosts, suggesting a limited potential role as a vector.

*Anopheles maculipennis* s.s*.* and *A. messeae* were rarely reported in Provence [214,215]. Moreover, the females of these two species are mainly zoophilic, meaning that they prefer large mammals as hosts for their blood meals, so they only bite humans when in close contact, thereby acting as vectors. *Anopheles messeae* is susceptible to high temperature and low humidity [211], explaining its weak density in Provence contrarily to lowlands of the French Alps [222].

#### Outdoor anopheline maximum abundance coincides with the “season” of endemic intermittent fevers

In Camargue in the 1950s, a study showed that larvae of *Anopheles maculipennis s.l.* were collected throughout the summer but peaked from late July to mid-August; in October, they became scarce and then disappeared [223]. During the same period, the overwintering adults were very abundant in the horse shelters, dog kennels and rabbit hutches. Collects of *A. atroparvus* made in 2005 resulted in the capture of extremely rare individuals [51], with a peak in October and November [53]. But before its decline, this species was observed to be quite abundant until September (study of 1943) [63]. In summary, these data suggest that outdoor anopheline imagos were present significantly to mid-June to September, after, it was principally found indoor overwintering *A. atroparvus* but they were, however, rare in the houses*.* However, as the temperatures of the 20^th^ century was higher than those of the period analysed, the time of presence of outdoor imago mosquitoes should just be a little more limited. *Anopheles hyrcanus* is currently the main potential vector of malaria in the Camargue area. Its mean annual dynamics have shown that their populations began increasing in the middle of June, reaching a peak near the middle of August and decreasing drastically in the middle of September [51,53,55]. None of these samples were collected during winter. *Anopheles melanoon* presented similar dynamics, although total mosquito numbers were very different and that decreased earlier [53]. However, environmental changes, mainly caused by anthropogenic practices, have had an impact on inter-annual variations in the abundance of mosquitoes [53]. For example, in Camargue, the pattern of population dynamics of *A. hyrcanus* and partially those of *A. melanoon* was related to the condition of the rice fields [53].

As already mentioned both the seasonality of endemic intermittent fevers and the epidemic peak time were almost always observed in summer and early autumn. Therefore, the outdoor anopheline maximum presence observed from June to September coincides globally with the “season” of intermittent fevers. The relatively high number of endemic intermittent fevers mentioned in October might be explained by the length of the incubation period in humans. At a temperature of 18°C, complete *P. vivax* sporogony requires 30 days [74]. A mean temperature of 18°C was generally reached by mid-May (Figure 3); if 30 days were added for the sporogony and 15 days for the incubation in the human host, intermittent fevers should mainly be observed from July which corresponds to the data mentioned in historical records [25]. The complete development of the parasite in the mosquito was most often not possible when the sporogony began in mid to late September (Figure 3). Thus, with a mean incubation period in humans of 15 days, the last primary attacks should begin in late October to early November, but during this period, intermittent fevers were generally rarer, especially primary attacks; this pattern was most likely due to the scarcity of outdoor infectious anopheline individuals since the end of September. This finding also suggests that as it seems that latent primary infections and relapses were rare, for the last infections of the infectious season, there were few relapses and likely no indoor transmission. Concerning the latter data, historical texts do not mention the nuisances of biting insects since October; therefore, either indoor anophelines were few in number and/or they wintered in premises containing domestic animals on which they took their blood meals.

It has been proposed that in Finland, relapses of *P. vivax* malaria, besides being genetically determined by the specific strain, are induced by the bites of uninfected vectors [48]. In past Provence, it seems that the period of intermittent fevers coincides with those where the temperatures permitted the outdoor completion of the vector cycle. It also appears that the relapses became very rare outside of this period. This finding could be seen as an adaptation of the parasite, as all activity in the host stopped during the year when the outdoor temperature did not allow for complete sporogony.

#### Change in anopheline species composition and/or decrease of the number of efficient vectors

A decline in malaria vector densities could contribute to the decreased levels of malaria infection as reported in several countries [224]. Historical, political, social, environmental and technical factors, including agricultural changes, could induce variation in the abundance of anopheline populations, with possible consequences for human health. Thus, the decline in intermittent fevers in Provence has been suggested as due to a decrease in the number of mosquito vectors [19]. However, the variations and distribution of the anopheline populations present in Provence during the period analysed remain unknown. Recent studies have shown that in Camargue, the abundance of potential vectors has varied greatly over the last 70 years, a detail which could be correlated with important anthropogenic ecosystem modifications, including intense mosquito control, during this same period [53]. In this area, *A. atroparvus,* the past main potential malaria vector has become very rare, the same phenomenon of the disappearance of this species was recently described in the Netherlands [225,226]. Similarly, in the 20^th^ century, the disappearance of *A. labranchiae* from Spain and of *A. sacharovi* from Malta and Romania has be described (references there in [209]). In addition, epidemics have been the result of the invasion or the reinvasion of a highly efficient vector that finds a permanent or temporary suitable environment, as occurred with *A. gambiae* in Brazil and Egypt in the 1930s and the 1940s, respectively, or with *A. darlingi* in Venezuela in the first half of the 20^th^ century [123].

The population dynamics of potential vectors of malaria depend on many climatic factors (e.g., temperature, rainfall and season) and anthropogenic factors (e.g., water management in relation to landscape use and mosquito control activities) [53,211,212,227]. However, during the studied period, the only significant environmental changes in Provence were some drainage of marshes. To date, the hypothesis involving a decrease in the number of efficient anopheline vectors is contradicted by the analysis of historical documents; indeed, there is no indication implying that there were variations in the endemic level during the inter-epidemic years, suggesting the ongoing importance of both vectors and parasites even if qualitative changes have likely occurred. Moreover, as already mentioned even in the early 19^th^ century, short travels of infected farmworkers continued to play a role in the spread of malaria. However, the factors determining subtle variations in the density of anophelines are not all known. For example, a study conducted in 2005 in Camargue showed that at the end of July, the dynamics of both the *A. hyrcanus* and *A. melanoon* species collapsed brutally in the two areas studied without any identified cause [53].

#### Refractoriness of Provencal anophelines to exotic strains of *P. falciparum* and *P. vivax*

The introduction of *P. falciparum* from Africa to South America and its transmission to human since the 16^th^ century constitute one of the best examples of historical parasite-vector co-adaptation of tropical *Plasmodium* strains to local anophelines, even if they are also tropical [228]. It is assumed that, before their “eradication” in 1976, continental *P. falciparum* strains were adapted to European anopheline vectors [3,229-231]. However, the continental species of the *A. maculipennis* complex including *A. atroparvus,* and even all European *anopheline* species*,* have also been reported as refractory (or essentially refractory) to both Asian and African *P. falciparum* but competent to tropical *P. vivax* strains (references in [51,213,232]). Among the anopheline species found in Provence, only *A. plumbeus* can significantly transmit tropical *P. falciparum*[221]. However, it would seem that in natural conditions and even if it is only with a low frequency, French anophelines may be involved in the transmission of tropical *P. falciparum.* Indeed, in France during the World War I, quite a number of autochthonous cases of malaria were recorded in areas where malaria had disappeared but where colonial or indigenous troops were stationed, demonstrating that local mosquitoes can be vectors for tropical *Plasmodium* including, although rarely, *P. falciparum*[8,233,234]. However, compared to the thousands of cases of exotic malaria, new epidemics involved only few indigenous cases [165,235,236]. Similarly, although on a smaller scale, to the south of Paris, where thousands of Senegalese, North African and Malagasy prisoners were grouped in June 1940, malaria cases were diagnosed in late July to late October in the local population, including two attributed to *P. falciparum*[237,238].

Since the end of the 1990s, isolated autochthonous cases caused by *P. vivax, P. falciparum* and *P. ovale* have been reported in Western European countries, such as Spain, Italy, Germany, Greece and France [239]. Among these cases, two instances of autochthonous transmission of *P. falciparum* were suspected in the western Provence coast in spring and summer 2006 [240], supporting the idea that French anophelines could transmit tropical *P. falciparum* parasites. Concerning one of the two cases, the neighbourhood included families of Comorian descent, in which members recently travelled to Comoros, where malaria is endemic. Entomological surveillance carried out four weeks after he became ill failed to find any *Anopheles* mosquitoes; however, the presence of urban anophelines such as *A. plumbeus*, which may transmit malaria, cannot be excluded.

This short analysis demonstrated that French anophelines are not (totally) refractory to tropical strains of *P. falciparum.* However, in the past, the possibility of transmission should be very low, although because of the means of transport, the long duration of voyages for the soldiers, and the persistence of *P. falciparum* in the body, few still had to be infected by this species on arrival in mainland France. For example, during the World War I the majority of patients, who, in Macedonia, presented with high levels of *P. falciparum* parasites in their blood, were observed to have almost only *P. vivax* after their arrival in France [241]. In addition, in the 20^th^ century, French anophelines also had difficulty transmitting exotic *P. vivax* strains. For example, the return of many soldiers of Macedonia (mainly infected by *P. vivax*) does not seem to increase the intensity of the local endemic in Provence and in neighboring regions [6,105,242-244]. Similarly, in the 19^th^ century, the return of Napoleonic troops and later those of soldiers from Algeria did not seem to have a health impact, unlike the traffic over short distances of individuals infected with strains of Provencal parasites. However, it is not known whether this characteristic of the anophelines of at least Provence was already present in the 18^th^ century. Indeed, the hypothetical acquisition of refractoriness in the late 18^th^ century could be one of the factors involved in the rapid decline of the great epidemics.

The study of the causes of the acquisition of refractoriness is of great interest because it could help to significantly reduce the impact of malaria in the world. The potential efficient anopheline vectors were known to exist in regions that were not malarious, although they could have been, given the climatic and ecological conditions. This “anophelism without malaria” was considered a puzzle and a paradox [245,246]. The Provence region currently presents this type of situation, as potential malaria vectors could be very abundant in some places [247-250]. However, this case did not apply during the analysed period as suggested by the persistency of a high to relatively high level of endemicity. The disappearance of malaria in Provence could be the result of a long and slow process that started in the late 18^th^ century during which the inability of potential vectors to transmit *Plasmodium* could have played a role. Putatively, in this mechanism, *Wolbachia* would have played a role. *Wolbachia* are intracellular and endosymbiotic bacteria that infect approximately two-thirds of insect species along with numerous other arthropods. Moreover, they are potent modulators of pathogen infection, and although *Anopheles* mosquitoes are naturally uninfected with these bacteria, artificial infections have shown that *Wolbachia* strains reduce the level of *P. falciparum,* although in contrast, a *Wolbachia* strain enhances oocyst density of *P. berghei*, the model murine malaria species [251-253]. Infection with *Wolbachia* can protect mosquitoes against *Plasmodium*-induced mortality in a natural system [253]. Nothing is known about the mosquitoes in the past and, although this is highly speculative, if *Wolbachia*-infected *Anopheles* had existed in preceding centuries, this point could lead to a decrease in the number of very infectious mosquitoes and so contribute to the disappearance of malaria in some areas. Furthermore, virulent *Wolbachia* strains significantly reduce the life span of their host, which can also have a dramatic effect on reducing pathogen transmission [251]; in other words, uninfected *Anopheles* could be positively selected relative to those that were contaminated. Moreover, some of the strains and/or genetic variants of *Plasmodium* may have been preferentially transmitted.

### Analyses of possible implication of climatic and physical conditions

#### Climatic factors and epidemics

Malaria is a complex disease, and its transmission and prevalence is influenced by many factors including meteorological conditions (temperature, rainfall and floods, which in turn depend on surface water, atmospheric moisture and wind). Weather affects the malaria incidence due to its effects on host/vector/parasite relationships (population dynamics of both anopheline vectors and *Plasmodium* species, survivorship and the development of the malarial parasite inside the mosquito vector – gonotrophic cycle – and transmission rates to human, and also human behaviours and agricultural practices) [102,254,255]. Sallares [33,188] considered that the evidence from Italy strongly supports climatic explanations for malaria epidemiology in southern Europe in the past. In a preliminary analysis, in western Provence, the years with malaria epidemics were observed to be significantly warmer (Student’s t-test for annual means, p-value = 0.0176), but not significantly more rainy (Student’s t-test, p-value = 0.7667). Thus, the influence of temperature and precipitation must be developed more precisely below. Noted in all the statistical analyses, the number of epidemics over the three studied areas has been grouped together.

**Figure 5:**
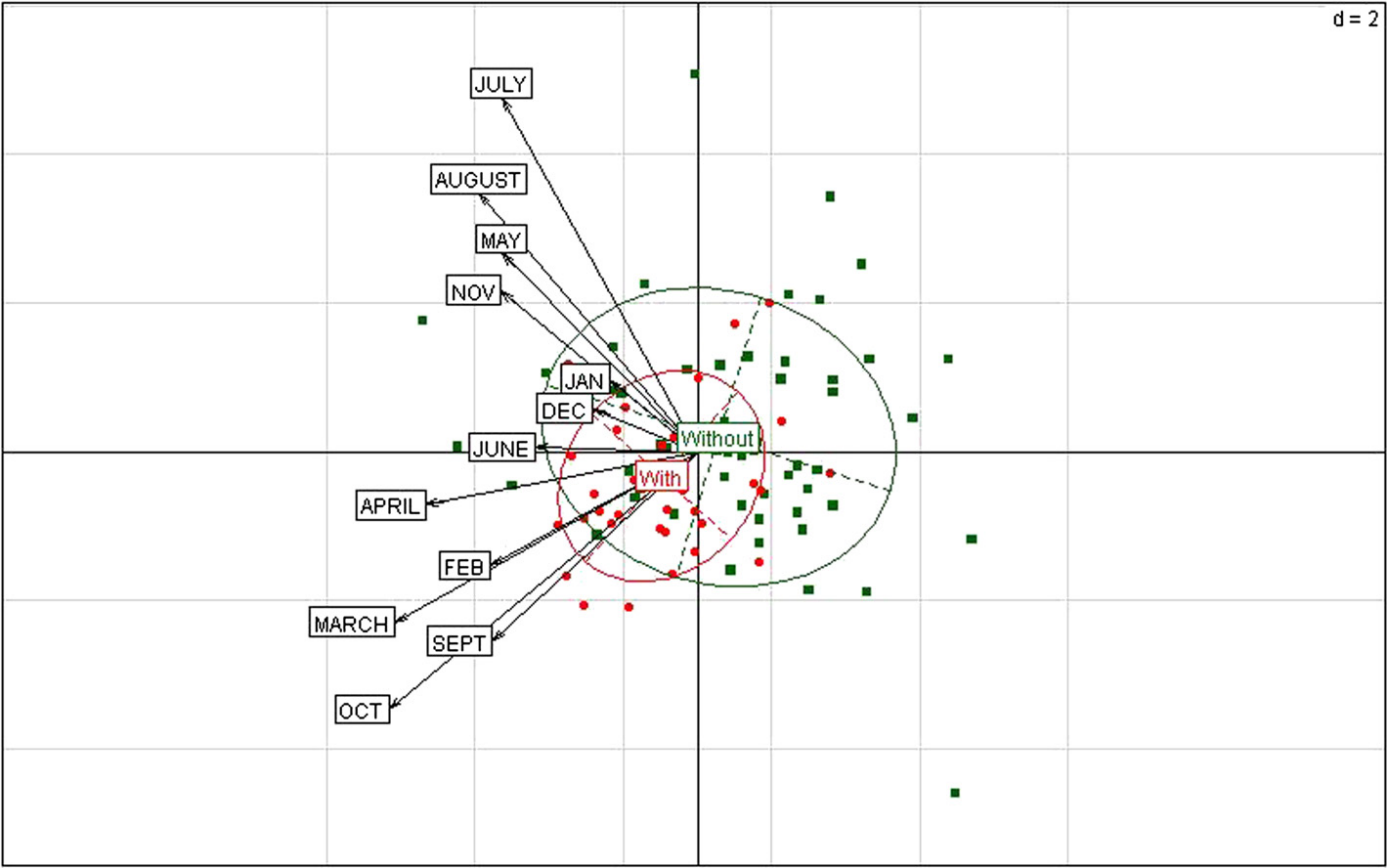
**Principal Component Analysis for monthly mean temperatures, with the binary epidemic factor as supplementary variable.** For November and December, the mean temperatures of the previous years were used. On the one hand, the horizontal axis, accounting for 17% of the total variance, gives a temperature gradient. Hence, the more a year is situated on the left, the warmer it is globally. On the other hand, the vertical axis, accounting for 13% of the total variance, permits to identify the years with warm intermediate seasons (situated at the bottom of the graphic). Years with and without epidemic are indicated with red circles and green squares, respectively. Thus epidemics were observed in years that were generally warmer, especially for intermediate seasons. Moreover, years with epidemic seem to keep a consistent profile with respect to monthly temperatures, since the associated ellipse is smaller.

In Marseille, from 1745 to 1850, the mean annual temperature was 14.1°C (6.4°C in January and 22.0°C in July). Even during the coldest summers of the period from 1745–1850, the sporogony of *P. vivax* should be always theoretically possible, e.g., the coldest summer was that of 1843, with mean temperatures for June, July, August and September of 17.7, 19.1, 14.4 and 21.8°C, respectively [256]. In contrast, analysis of daily temperatures demonstrated that the sporogony of *P. falciparum* in some years was hardly compatible with mosquitoes’ longevity at that time. Generally, warmer weather will enhance transmission directly because as temperatures climb, so will the rate at which mosquitoes develop. Adult anophelines will feed more frequently on blood (so they will pick up and transmit the infection faster), and malaria parasites will develop quicker in the mosquito. Figure 5 illustrates this phenomenon: years with epidemics tend to be warmer, especially for intermediate seasons (spring and autumn). Unfortunately, the principal components are poorly informative (providing 30% of the total inertia), but the result can be validated by statistical tests for March, September and October monthly mean temperature; they are significantly warmer during the years with epidemics (Student’s t-test, p-value_March_ = 0.0006, p-value_September_ = 0.0827 and p-value_October_ = 0.0048). Warmer temperatures in early spring could play a positive role in the development of mosquito larvae, whereas hot temperatures in September and October prolonged the period of infectious bites and therefore increased the risk of infection and multiple infections. Besides, during epidemics, the average temperature during the months of July and August was not significantly different from the average over the other years (Student’s t-test, p-value_July_ = 0.8493 and p-value_August_ = 0.3956).

**Figure 6:**
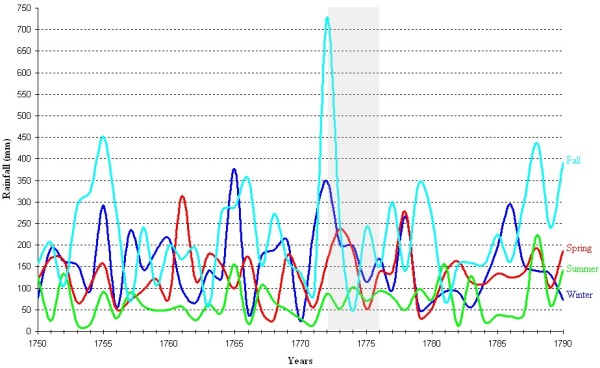
**Total rainfall by season at Marseille.** The last great epidemic period (1772 to 1776) has been underlined in grey. The seasons were interpreted as follows: winter (December of the previous year, January and February), spring (from March to May), summer (from June to August) and fall (from September to November). For reasons of readability, only the period from 1750 to 1790 is shown.

Concerning precipitation, the Mediterranean climate of western Provence is subject to the following rhythm: two dry seasons, including a brief period in late winter and a very long and pronounced period in summer; two rainy seasons, one in autumn with abundant rains or even torrential, and another less pronounced in the spring (Figure 3). One of the characteristics of the Mediterranean climate is that the warmest period (summer) is the one that is the least rainy; however, in the past, as observed in several other malarial Mediterranean areas, the rainfall deficit could be compensated for by the presence of large wetland areas. In Provence, the summers are hot and dry due to the rise of subtropical anticyclones, which can be interspersed with sometimes violent thunderstorm episodes. However, great variations could be found according the years (Figure 6). At Marseille, from 1749 to 1850, the mean annual rainfall was 517 mm even if great variations could be found from year to year (274 mm and 1316 mm in 1817 and 1772, respectively). The precipitation occurs mainly in autumn (41.6% of the annual rainfall); the two rainiest months of the year were October (95 mm) and November (78 mm). As already mentioned, the driest season is the one that is the hottest; 12.3% of the annual rainfall fell in summer, with the drought reaching its peak in July (15.6 mm) and 25.6 mm and 22.6 mm for June and August, respectively. Rainfall during winter and spring represented, respectively, 24.9 and 21.2% of the mean total annual precipitation. Rainfall influences the vector population by providing surface water for breeding places, which is determinant in non-marshy areas but also for the maintenance of ponds. Frequent precipitations play a significant role in generating moisture and therefore suitable conditions for adult vector activity and survival [257]. Of note, in historic Provence, heavy rains were also frequently considered as responsible for epidemics; this detail concerned principally those rains of the previous autumn and winter [25]. Interestingly, statistical analyses showed that during the years with epidemics, significantly more precipitation was observed in December of the previous year (Student’s t-test, p-value_December_ = 0.0237). According to contemporary witnesses, rainy summers were always fatal and caused numerous intermittent fevers [15]. High rainfall in July and August not only permitted the *Anopheles* vector to always find spawning sites but also facilitated a moist atmosphere for the imagoes. However, no such relationship can be confirmed by statistical analysis (Student’s t-test, p-value _July_ = 0.9811 and p-value _August_ = 0.7837), but the level of these rainfalls could also have increased the level of endemicity, a point that has not been analysed in this study.

**Figure 7:**
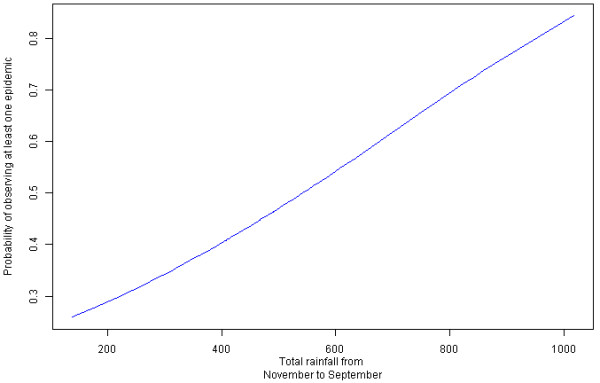
**Theoretical probability of observing at least one epidemic according to the total amount of precipitation.** The generalized linear model with a Poisson error distribution was used to compute the fitted probabilities. The fitted values are plotted against the amounts of precipitation (from previous November to current September). The more it rains in the previous winter, current spring and summer, the more probable epidemics occur.

Extreme climatic events could also govern the transmission of malaria, e.g., frequently malarial epidemics have followed natural disasters (such as floods or earthquakes) [116,141]. The major natural disasters that took place in western Provence during the period 1745–1850 were floods and they are principally due to those of the Rhone River. The hydrological regime of this river basin is complex with a great diversity in the formation of floods and of their progress. However, a majority of observed floods of this river from Avignon to the Mediterranean Coast are due to precipitation which had a Mediterranean origin [258], which could explain how inundations could also be observed in the Berre area. In western Provence, floods were generally observed in winter and spring and lasted one or two months [140]. Floods in later winter and in spring increased the level of the water table and so could drench the ground and make it more suitable for mosquito breeding purposes in the subsequent late spring and summer. Interestingly, a correlation has been found between years with epidemics and the total amount of precipitation during the previous winter and spring (from the prior November to the current March) (Student’s t-test, p-value = 0.0396). This finding completes the previous statistical analysis that had only revealed the influence of rainfall in later December on epidemics. Moreover, a strong correlation is observed between heavy rains during the following months (April to September) and floods during the same period (generalized linear model, p-value = 0.0048). The effect of floods from April to September, even if they lasted less than a month, could persist for some weeks and so created spawning sites. The permanency of surface water then permits several generations to develop and allows for the maintenance of adequate atmospheric moisture. Thus, a strong correlation has been found between years with epidemics and the total amount of precipitation during the previous winter, spring and summer (from the previous November to the current September) (generalized linear model, p-value = 0.0027). Rainy years are more favourable for epidemics, where the term year is defined in a convenient sense rather than as a calendar year. Figure 7 plots the theoretical probability of observing at least one epidemic in the studied region, according to the total amount of precipitation (from previous November to current September). This result matches with the usual consideration that the great epidemic period that raged in several parts of Provence was the consequence of the very rainy autumn and winter of 1772 and spring of 1773 [25] (Figure 6).

**Figure 8:**
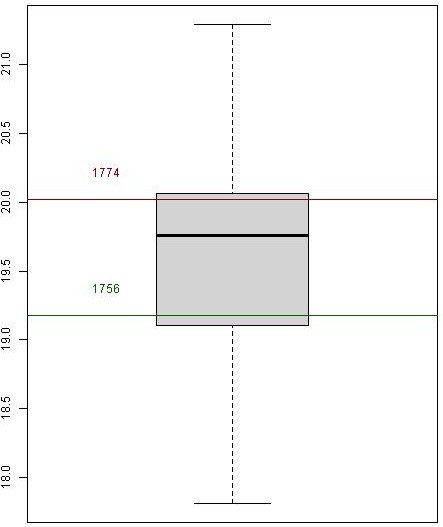
**Boxplot for the mean of the temperatures from July to October.** The box plot gives a representation of the concentration of the mean temperature (from July to October) over the period 1745 to 1850. The years 1774 and 1756 are pointed out. The middle 50% of the years fall inside the grey box, whereas the 25% warmest and 25% coldest years are above and below the box, respectively. The black line in the rectangle corresponds to the median temperature.

Frequently in historical documents, a link was made between heat waves during summers and increases in fevers. For example, in 1774, the “extreme and unbearable heats” felt in early August were incriminated in severe epidemics that followed in the region of Arles [25,259], whereas the mean temperature at Marseille during this month was approximately 24.1°C. When extreme events were observed just before or during an epidemic, they could be frequently considered as causative (or facilitating); however, during some years with very high summer temperatures no epidemic consequences have been mentioned, as e.g. in 1756. This is however not surprising because it has been previously shown that summer temperatures are not relevant in explaining epidemics, but rather those of September and/or October provide predictive information. In other words, the months of July and August months can be very hot but become deleterious only if hot temperatures are observed during the following weeks. This point is confirmed by the analysis shown in Figure 8; in the reported year of 1774, the mean temperature from July to October reached 20°C (73%-quantile), whereas 1756 could be considered as rather cool with 19.2°C (corresponding to a 29%-quantile).

Hot rainy seasons are generally linked to malaria epidemics, but paradoxically, it has also been mentioned that in some areas of Africa and Asia, severe drought has precipitated severe epidemics [123], as the vectors can breed profusely in the numerous pools formed in the river beds when the flow decreases and in artificial reservoirs. In western Provence, long periods of drought were frequently observed; in the most extreme cases, there was no precipitation during one of the summer months (46 months in total during the period analysed) with even a total absence of rain during two consecutive months, as observed for 5 years. Moreover, depending on the year, the drought could begin in spring and last until September. However, in western Provence, no such relationship was observed in statistical analyses. The drought during the hot season did not correlate with epidemics Chi-squared test of independence, p-value_JA_ = 0.1701, p-value_JJA_ = 0.3671, p-value_JAS_ = 0.4524). In Provence, the impact of malaria decreased during dry periods, this could suggest that the number of anophelines that could use artificial nesting sites, such as *A. plumbeus,* were few in number and/or were not effective vectors.

The majority of winds were considered to have their own pathophysiological effects, usually with deep antagonism between the northern winds – considered healthy, particularly in terms of fevers – and the southern winds, supposed to induce or increase the risk of malaria [260]. Moreover, specific local winds, often of thermal origin, could also be incriminated by indigenous populations, especially if they constituted downwind from a malarious area such as a wetland. In Provence, northern winds were frequently conceptualized to have a beneficial role [25,84]. However, the health considerations of wind were too numerous, too geographically localized and especially too contradictory to be analysed in this article. But interestingly, a recent report stated that in Kenya wind direction in relation to breeding site location may be a key element in determining the location of malarial hotspots [261].

Statistical analyses demonstrated that in western Provence, for a given year, a malaria epidemic was more frequently observed when a malaria epidemic had raged during the previous year (Chi-squared test of independence, p-value = 0.0535). During an epidemic a great number of individuals could be infected but due to the high level of endemicity in Provence, the global number of infected people should not vary much. Thus, if the variations should not really be quantitative, they should be qualitative, i.e., during epidemics infected individuals with acquired immunity became sick and especially there could be changes of virulence in the *Plasmodium* strains. Therefore, the year following the first epidemic, the most critical element may not be the weather but rather the presence of a large number of people infected by parasites inducing severe clinical signs, even in individuals who had previously developed acquired immunity against the “endemic *Plasmodium* variants”. It can also be assumed that the severe symptoms could appear only when one individual was infected with two or more types of strains or variants; the presence of a large number of people infected at the beginning of the summer transmission period would increase the risk of plasmodial co-infections. The importance of the previous year on the malaria impact has already been demonstrated in Europe, but this finding concerns only weather and mainly temperature. For example, in the French Alpine valleys, the mean temperature recorded in June between 1828 and 1900 was found to partially correlate with the number of deaths in the following year [218]. However, in western Provence, no effect of the June temperature on epidemics in the following year has been demonstrated (Student’s t-test, p-value_June_ = 0.3626). In Finland, correlations were observed between the summer temperature and malaria deaths during the following year; it has been suggested that summer temperature would regulate the density of vectors in the adult stages [101]. The same type of phenomenon was also observed in western Provence. As stated before, summer temperatures of the current year were not correlated with epidemics, but high summer temperatures during the previous year (from July to September or October) significantly favoured epidemics (Student’s t-test, p-value_JA_ = 0.1428, p-value_JAS_ = 0.0412, p-value_JASO_ = 0.0144). Yet note that the mean temperature from July to October during the previous year correlated with temperatures of the same period of the following year (linear model, p-value_JASO_ = 0.0706). Hence, it is difficult to show whether the temperature is a delayed or immediate factor. In the coldest regions of Provence, temperatures of the previous year could play a role because they permit transmission of *P. vivax* strains with a long incubation period; therefore, the clinical consequences were only or principally observed during the following year. However, in Provence, epidemiological analyses suggest that this type of strain did not play an important role; it is more likely (and a more important factor) that hot summers promoted an increase in the number of mosquitoes during the following year. In historical marshy areas of England, statistical analyses suggested that the malarial epidemics were partly due to precipitation during the same year [132]. In contrast, Bellincioni (1934) [262] found a very close correlation at Grosseto (in a marshy area of Italy) between rainfall levels in the months from September to May and the frequency of malaria in the following summer and autumn over a period of thirty-two years in the beginning of the 20^th^ century. According to him, the connection was in terms of fluctuations in the level of the water table, which must be linked to fluctuations in the size of the anopheline populations. The correlation between rainfall levels in the months from September to May and the frequency of malaria in the following year in western Provence was tested (generalized linear model, p-value = 0.0898), but it was much less relevant than the correlation found with rainfall levels in the months from November to September.

**Figure 9:**
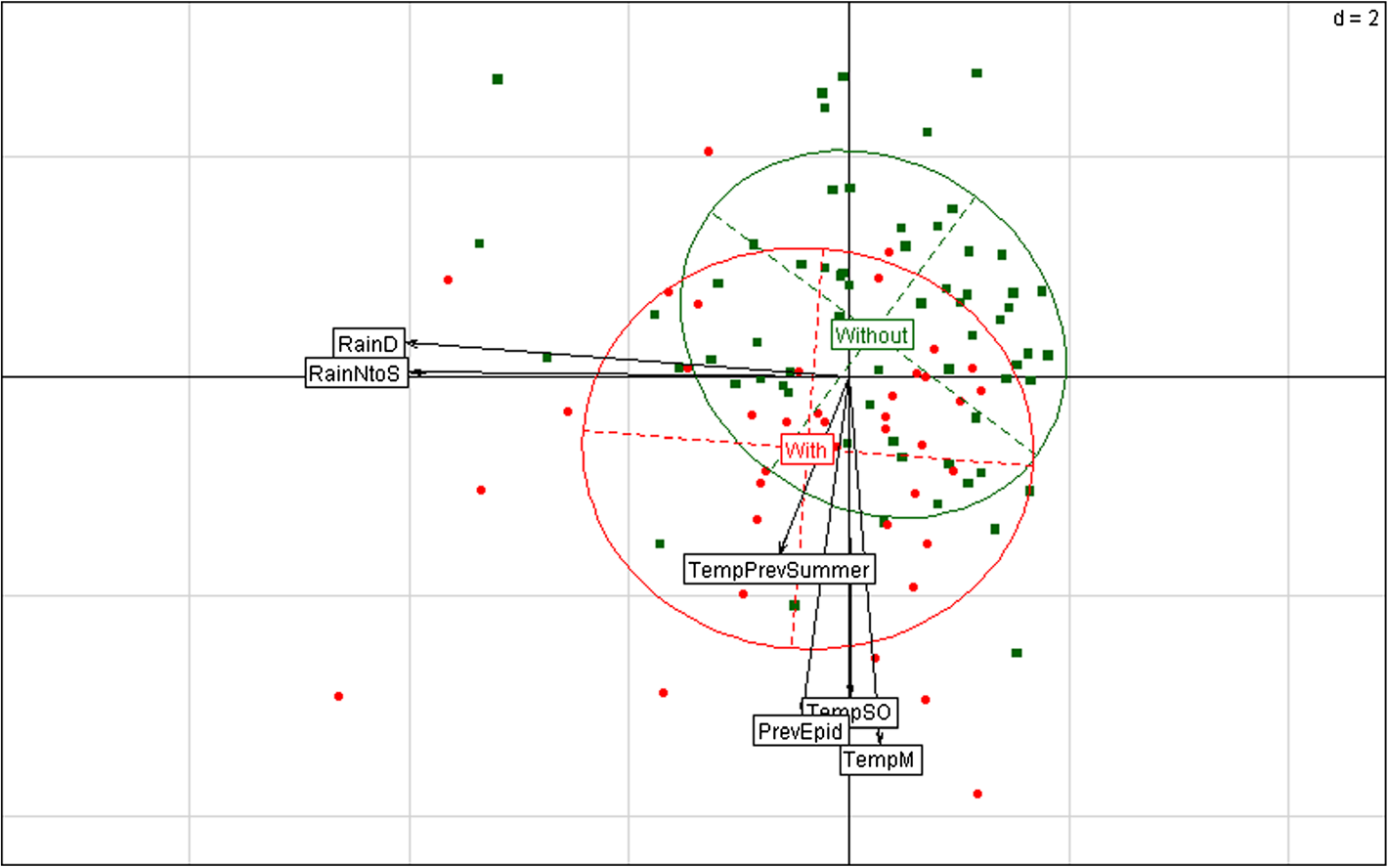
**Multivariate analysis to illustrate the interrelationships between the favourable factors.** Abbreviations for variables: RainD, rainfall during the December month of the previous year; RainNtoS, rainfall from November to September; TempPrevSummer, mean temperature of the summer of the previous year; TempM, mean temperature of March; TempSO mean temperature of September-October; PrevEpid, presence of an epidemic during the previous year. On the one hand, the horizontal axis, accounting for 27.2% of the total variance, gives a precipitation gradient. Hence, the more a year is situated on the left, the more rainy it is globally. On the other hand, the vertical axis, accounting for 26.4% of the total variance, summarizes the factors due to temperature and to the presence of an epidemic the previous year. The binary epidemic factor is used as a supplementary variable. Years with and without epidemics are symbolized by red circles and green squared symbols, respectively. Thus epidemics were more frequently observed in years that were warmer and rainier (the convenient months).

Statistical analyses have demonstrated that three types of factors could have played a decisive role in the emergence of epidemics. These factors are the relatively high temperature in early spring (principally in March) and in September-October, rainfall during the previous winter (principally in December of the previous year) and even during the period from November (of the previous year) to September, and the presence of an epidemic during the previous year. Statistical analysis was performed to determine if there were interrelationships between these factors. The multivariate analysis with mixed quantitative variables and factors allowed to distinguish two types of independent factors: the first one related to rainfalls and the second one related to high temperatures and to the presence of an epidemic during the previous year (Figure 9). This point could suggest that the factors that concerned principally the vectors (rainfall) would be independent of those being more favourable to parasites (temperatures and epidemic(s) during the previous year).

#### Search of explicative factors of the epidemic breakpoint

As previously shown, analyses of the historical data allowed to date the epidemic breakpoint in 1776. Up through 1776, severe epidemics ravaged large areas, and after this date, epidemics were rarer, much more localized and generally benign, at least for the natives. Relatively moderate upsurges of epidemics was observed in the early 1810s but were generally very localized, not serious at least for the indigenous populations and mostly linked to the digging of the canal from Arles to Bouc. As climatic conditions played a role in the emergence and perpetuation of epidemics, it was examined whether there were changes in these epidemics before and after the breakpoint period.

**Figure 10:**
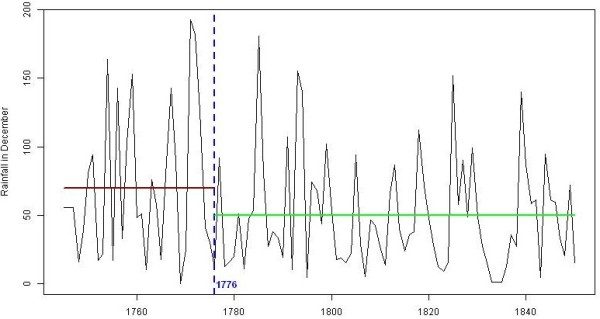
**Rainfall in the December month before and after the breakpoint year (1776).** The mean monthly total rainfall before and after this date is indicated by brown and green horizontal lines, respectively.

In western Provence, in terms of rainfall, significant changes were observed before and after the breakpoint period only for October (Student’s t-test, p_October_ = 0.0245) and December (Student’s t-test, p_December_ = 0.0713). October months were less rainy before the epidemic breakpoint, whereas December months were more rainy. As no significant role for October precipitations in the epidemics was found, then it may not be at the origin of the breakpoint. On the contrary, statistical analyses have demonstrated that strong precipitations in December significantly favoured epidemics. As illustrated in Figure 10, the sudden and dramatic decrease of precipitation during this month just before 1776 may partially explain the breakpoint. Interestingly, in sub-Saharan semi-arid African countries, rainfall anomalies are the main determinant of epidemic outbreaks [263].

**Figure 11:**
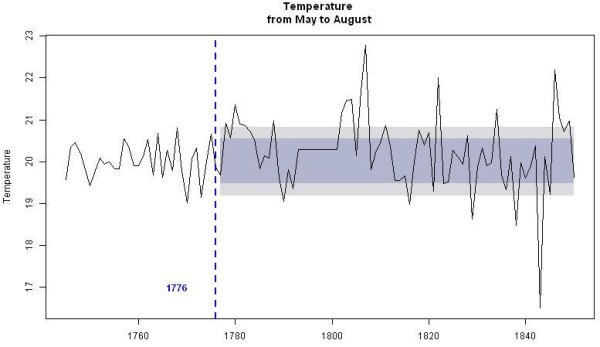
**Study of the fluctuations of the mean temperature from May to August using a Gaussian white noise model.** The year 1776 was the epidemic breakpoint. An Arima model was used to plot prediction intervals at confidence levels of 80% and 95% in light grey and grey, respectively.

Statistical analyses of monthly mean temperatures did not reveal a significant change before and after the breakpoint year, except for May (Student’s t-test, p_May_ = 0.0149), which was warmer after the breakpoint. However, previous analyses did not show that the mean May temperatures had a significant role in the increased epidemics, thus the increase of temperature during this month is likely not be at the origin of the breakpoint. Besides, the mean annual temperature during the period 1745–1776 was similar to those of the period of 1777–1850 (~14.1°C) (Student’s t-test, p-value = 0.2659). However, analyses of the extremes of mean annual temperature in Marseille from 1745 to 1850 revealed that both the maximum and minimum mean annual temperatures occurred after the breakpoint year: 15.5°C in 1846 and 12.9°C in 1843, respectively. Therefore, it could be interesting to analyse the variations of temperature according to the period. Thus monthly mean temperatures were significantly more variable since 1776 for the months May to August, and with less intensity for September (Fisher’s test, p_May_ = 0.0142, p_June_ = 0.0091, p_July_ = 0.0533, p_August_ = 0.0013, p_September_ = 0.0969), i.e., in other words, during the months when the sporogony of *P. vivax* was theoretically possible. Then, an AutoRegressive Integrated Moving Average (ARIMA) model was constructed for the mean temperature from May to August, including only the years before the breakpoint. Gaussian white noise is a convenient model for this series (Jarque-Bera test, p-value = 0.871; Shapiro-Wilk test of normality, p-value = 0.7426; Ljung-Box test, p-values > 0.1967 for any lag) and for even the best ARIMA model according to the Akaike’s information criterion (AIC) [62]. In Figure 11, this model has been used to plot prediction intervals at a confidence of level 80% (respectively 95%) in light grey (respectively in grey) for the years following the breakpoint. By comparing with the observed temperatures after the breakpoint, it can be concluded that monthly mean temperatures are clearly more variable after this point of rupture. Indeed, during the months when the sporogony and/or outdoor transmission were possible, 43.2% of the years from 1777 to 1850 (resp. 28.4%) have an average temperature higher or lower than the prediction intervals at confidence level 80% (resp. 95%), inferred from the previous period, which is highly significant (Chi-squared test of goodness-of fit, p_80%_ = 5.7 × 10^−7^ and p_95%_ < 10^−8^). Note that inferring missing values from 1793 to 1801 by the associated monthly mean tends to reduce the variability after the breakpoint.

Nevertheless, this increase of monthly mean temperatures variability is hard to connect with mosquito or larval development; it could be hypothesized that mosquitoes would have difficulties to adapt to temperature variations from one year to another. Actually, in order to seek out more accurately the effect of temperature variability, daily temperatures are required. Actually, two datasets are available for daily temperatures: the first one covers years from 1745 to 1787 and temperatures were recorded twice a day (at 7:00 am and at 2:00 pm), whereas the second one covers the successive period, including successive months of missing data, and containing three measures per day (the mid-measure being recorded at 3:00 pm). The study of daytime temperature has been restricted to the first period, which surrounds the putative breakpoint. Thus absolute fluctuations between the daily temperature average and the global mean temperature of the month level were studied. Significantly larger temperature fluctuations were observed since 1776 for the months December (of the previous year), and for April, May, August of the current year (Student’s t-test, p_April_ = 0.0299, p_May_ = 0.0004, p_August_ = 0.0123, p_December_ = 1.9 × 10^−6^). Actually, temperatures are also more variable in April, May and December (Fisher’s test, p_April_ = 0.0356, p_May_ = 0.0051, p_December_ = 0.0006).

In summary, after the breakpoint, the months with the most extreme temperatures are April, May, August and December and those with more variable temperatures are April, May and December. Even if theoretically in May, the mean temperature allowed the *P. vivax* sporogony, most of the anophelines were still in the larval stage. So, in April and May, the extreme value of temperature and their variability, if they played a negative role in the emergence of epidemics, would have acted on the larval development. Extreme temperatures in August could act both on parasites and vectors that could result in a shutdown or significant reduction in transmission during this month which should also have repercussions on the number of infected mosquitoes in September and October. In December, more extreme and variable temperatures were observed after the breakpoint, but no link was made between epidemics in western Provence and cold temperatures [25]. However, during winter, alternating cold days and other hottest oblige the females of *A. atroparvus* to emerge from their hibernation reducing their life expectancy which will have an impact on egg laying and therefore on the number of imagoes during the next summer.

It is probable that temperature fluctuations observed after the breakpoint might substantially alter both the survival of the vector and the development of malarial parasites in mosquitoes, thus influencing malaria transmission rates. This hypothesis finds significant support in the so-called Kaufmann effect (1932) [264], according to this, the biological processes are affected by fluctuation of temperatures; this effect has long been recognized, and the influence of monthly, weekly and daily temperature variations has been established in a range of other host/vector/parasite interactions [265]. The key *Anopheles*/*Plasmodium*-related traits that together determine malaria transmission intensity (i.e., parasite infection, parasite growth and development, immature mosquito development and survival, length of the gonotrophic cycle and adult survival) are all sensitive to daily variations in temperature [266,267]. In western Provence, the high temperature fluctuations after the epidemic breakpoint could be one of the explanatory factors of both the cessation of great epidemics and of the beginning of a decrease in the endemic level. In addition, according to the model of Mordecai *et al.*[73], the optimal malaria transmission is at 25°C, whereas the transmission would drop dramatically at temperatures >28°C. However, in western Provence, if daily mean temperatures higher than 25°C have been observed relatively frequently from June to August, then temperatures higher than 28°C were relatively rare.

#### Altitudes and downtowns

As a general rule, the temperature rate decrease per 100 m in the International Standard Atmosphere is 0.65°C [268]; therefore, the relationship between altitude and decreased malaria transmission is well known. In France, malaria was generally absent at altitudes higher than 400–500 meters. The altitude of the towns and villages of the studied area are mentioned in Additional file 3[269,270]. In this zone, most of the people lived at an altitude less than 50 m, so no altitude correction was necessary. In addition, the majority of rich farmlands (and of the swamps) were at an altitude less than 10 m.

During the 19^th^ century, in the Alpine valleys of southern France (altitude approximately 300–500 m) malaria was endemic and life expectancy particularly shortened [218]. However, in Upper Provence (altitude > 300–400 m) malaria endemicity was generally absent. When very serious epidemics raged in these areas, which rarely happened, they were most likely the result of infections that would have occurred elsewhere, and usually they did not last, perhaps due to a combination of two factors (a lower number of efficient vectors inducing a weak level of multiple infections and lower temperatures than those in lower Provence). For example, in 1775, while severe malaria epidemics raged in various areas of low Provence and Languedoc, less severe episodes are mentioned in medium-altitude areas, such as in a commune near Sisteron located outside and northeast of the study area (minimum and build-up area altitudes: 471 and 600 m, respectively). In this village, a relatively important number of sick and dead people were mentioned from February to March (a peak epidemic period never observed in the studied area), especially among farmers, which suggests both that the infections were occurred during 1774 and that there was no diffusion of the epidemic into the population, as the document is dated August 1775 [25]. Even if in this village, situated at approximately 75 km from Marseille, the temperatures were not known, and the mean temperature of Marseille in July and August 1775 corrected for altitude was 18.8°C. Similarly, in 1786–87, whereas severe epidemics were mentioned in towns of Arles and Berre areas, a higher number of malaria episodes than usual were observed in 1787 in various villages around Manosque, located south of the village mentioned above (build-up areas altitudes: 300–400 m). Some intermittent fevers were mentioned in the beginning of spring but they principally raged since the end of June and ceased during the first colds [25]. Before 1787, no epidemic was mentioned in the surrounding area of Manosque; however, labourers of this region worked around Arles. This epidemic is most likely due to first contaminations in 1786 added to the favourable weather conditions in 1787; indeed, relatively high temperatures were observed from July to August (the temperature of Marseille corrected for altitude was approximately 21.4°C). In the case of Sisteron, indoor infections can be excluded because the epidemic that raged from February to March 1775 mainly affected men working in the fields. The infection therefore occurred the previous year, and the latency period seems to have been long enough. In addition, in 1775, there would have been no transmission despite the fact that the population should not have acquired immunity. Indeed because during summer, the number of infected people should be lower (no longer mentions of patients from this season) suggesting that in spite of favourable temperature conditions, the number of effective and/or infected vectors could be very low. In the case of Manosque, a village at half the altitude as Sisteron, if indoor transmission was excluded, there were also infections in the previous year which could explain the spring intermittent fevers, but the epidemic was only observed during the period where the outdoor transmission was possible. Moreover, the strains implicated in these two villages have particular characteristics and persist for only a few months in human hosts. These two examples demonstrated that malaria in areas close to the studied region but at higher altitudes could not spread or be maintained, and as the local population should not have acquired immunity, the temperature factors and the type and number of potential vectors seem to be the key parameters.

Within the studied area, endemic malaria occurred mainly in low-altitude areas and only occasionally in higher altitude spots, but contemporary physicians observed higher morbidity and mortality levels during epidemics of intermittent fevers in villages and houses placed on heights [20]. As the study of these epidemics can provide information on the conditions for spread of malaria and the immune status of the hosts, two examples are discussed below. In the studied region, the built-up area which has the highest altitude is Velaux (altitude of the town hall: 130 m). This village is around the Etang of Berre but without direct contact with the latter and its land was considered dry. In the 1780-90s, the village was regarded as the healthiest in Provence [24]. This assertion seems doubtful but it was perceived as such because it was very close to the very malarious areas of the Etang of Berre. As already underlined by Sallares (2002) [33]: *“to understand the ancient preference for elevated locations, it is essential to remember that it is only when the comparison is with lowland populations affected by endemic malaria that upland regions … in the past had an overwhelming advantage in terms of health”*. According to documents preserved until today, it seems that only one malarial epidemic raged in the village of Velaux during the studied period. During the years 1772–1777, as already mentioned, malarial epidemics raged in several parts of Western Provence, and in 1774, a physician noted that epidemic fevers (intermittent and continuous) ravaged Provence, including the village of Velaux [25]. In this village, fevers began in spring and in April out of 822 inhabitants, 160 were sick and more than 25 adults died. The mortality/morbidity rate was therefore higher than 16% not including the deaths of children, which could be high during malarial fevers. As fevers are generally very scarce in Velaux, the mortality rate could partly be explained by the fact that due most likely to relative altitude, most of the population was rarely in contact with *Plasmodium* and therefore the pre-immunization rate was low. In the first months of 1774, malaria transmission did not occur due to cooler temperatures; indeed, the mean temperatures at Marseille were 7.6°C (January), 7.2°C (February), 12.2°C (March), and 14.6°C (April). This finding demonstrates that sporogony was impossible for outdoor anophelines with the exception of the end of the April, but this possibility was incompatible with an epidemic during the same month. Thereby, if indoor transmission of sporozoites throughout the winter by semiactive hibernating mosquitoes was rare if not excluded, effective infected bites occurred in 1773, but beyond this village, as it was considered healthy and malaria episodes were not mentioned during this year. In 1773, there were malaria epidemics with high mortality rates in various parts of Provence [25]. Moreover, in the same year, according to a physician, in Arles, severe epidemic intermittent fevers have prevailed during the autumn, which was considered as rainy and cold [25] as confirmed by meteorological data. Indeed, in 1773, it rained more than average from spring to autumn and the mean annual temperature at Marseille was relatively low, however, was theoretically compatible with the sporogony of *P. vivax* and of *P. falciparum* from May to October and from June to October, respectively. Concerning the epidemic of Velaux in 1774, the long period of latency (more than five months) suggests *P. vivax* infections. The most parsimonious hypothesis with regards to the data presented above, is that inhabitants of Velaux have been infected at the latest during the month of October 1773. As this village is considered as healthy, the localization of infections is unknown; however, the same physician mentioned that in 1773, intermittent fevers (tertian and quotidian) were introduced in Marseille by “foreigners”; this approach leads to propose that two sources of contamination are possible: agricultural labourers of the village working areas of very low altitudes and foreigners. However, if the contamination conditions were those just described, epidemics should occur almost every year, which was not the case.

Another similar example can be found in a village closer to the Etang of Berre than Velaux, this is Saint-Mitre, which is located on a rocky spur with an altitude of the town hall of approximately 90 m. This commune was also considered as relatively healthy, despite the presence of ponds accused of being responsible for some cases of intermittent fevers [15]. However, it was once again a relative consideration because the health situation of this village was compared to others around the Etang of Berre. Indeed, the number of annual births and deaths were recorded from 1803 to 1850, and the number of death outweighs those of births during a fifteen-year interval [18,19]. In this community, the rate of endemicity was perceived as very low, which should correspond to reality, but during epidemics, high morbidity and mortality rates were mentioned. For example, during the malarial epidemic of 1828–1832, where the population incriminated a small pond located less than two kilometers from the village and at an altitude of 5.6 m, nearly half of the population was affected during the first year but mortality rate was very low during this year [19,80]. Depending on the authors, 50% to 96% of the population was sick during the epidemic episode [99,100]. During the years 1829 to 1832, nearly a quarter of the population of the village died, while in years without epidemics, less than 12% of the population died over a period of 4 years [18]. From 1828 to 1832 at Marseille, the mean temperature of July and August for each year was higher than 21.8°C, with the exception of 1829 (20.0°C). In a general way, the factors underlying the micro-epidemiology of malaria are not fully understood but include variation in distance from the nearest mosquito breeding site, wind direction, house construction features, human behavioural and genetic factors [261]. In the two villages, Velaux and Saint-Mitre, the most determinant factors were most likely the proximity with mosquito breeding sites (temporary and permanent marshes, respectively), altitudinal situation, endemicity in neighboring regions and (low) level of acquired immunity. The morbidity and mortality levels suggest that within an area of high endemicity, the populations living in a relatively higher altitude environment was less affected by endemic malaria, which would result in low pre-immunization rates and therefore result in a very high sensitivity level during epidemics. This result also implies that a large number of the inhabitants of these areas are rarely down in lowland areas during the transmission season, which is not mentioned in documents contemporary to the studied period. Similarly, in marshy areas of past England, the epidemics of 1779 to 1881 were said to harass the upland villages more than communities in adjacent valleys [59].

Furthermore, generally it was also observed a dropped impact of malaria in town centres, and even the centres can remain totally unscathed [2,29,75,271]. Three elements, taken together or individually, can generally explain this phenomena because urban environments could be inappropriate for anopheline vectors: the centre of the town is further from aqueous or flooded areas and/or surrounded by large walls and/or located for defensive reasons on a small elevation. In Provence, it was observed by contemporary physicians that intermittent fevers were very common and often took a character of malignancy in lower parts of the cities contrarily to the higher places of the same towns (e.g., in Arles [25,85] or Avignon [272]). These observations have been made during floods, but not always. Moreover, in endemic rural areas, a building that is surrounded by strong walls, such as a monastery, can be free of malaria even during severe epidemic episode in central France in the mid-18^th^ century [128]. In Provence, in the Montmajor Abbey, which was built on a modest limestone hill (43 meters) among marshes, the monks, who were likely protected by the surrounding walls and by the thick walls of the buildings themselves, rarely contracted the intermittent fevers that raged on the plain (generally 1 to 3 meters above the sea level, and most likely lower in the past) [85]. Moreover, the idea that the upper storeys of a house were safer and healthier with regard to malaria or to avoid mosquito bites was widespread both in Ancient Egypt [273] and elsewhere in premodern Europe [33]. Similar convictions were held by the inhabitants of Provence; e.g., according to a physician, in Arles, malarial fevers raged only on the ground floor or in the lower floors [85]. According to Sallares [33], it has been frequently observed that mosquitoes, being weak fliers, are reluctant to fly up to the higher storeys of multi-storey dwellings.

## Conclusions

Generally in the historical studies, the conditions of decline and disappearance of endemic malaria are analysed but not those of epidemic outbreaks. Western Provence was considered as a “country of fever”, where despite a high rate of malarial endemicity, very severe epidemics of intermittent fevers raged frequently with a high morbidity level usually associated with pernicious clinical signs but not always associated with a large number of deaths. In this region, severe malaria epidemics that plagued large areas stopped definitively to break out in the middle of the second half of the 18^th^ century (dated 1776), after the last major epidemic episode that had raged in a large part of Provence. Moreover, after this breakpoint, the decline of endemic malaria also occurred, albeit at different rates according to the areas, but by 1850, the incidence of malaria had become very low except in Camargue.

Epidemic malaria derives from particular interactions of vectors and parasites as well as various environmental and anthropogenic determinants. Therefore, in this study, the majority of the possible influencing factors have been analysed. Before the development of malaria can be controlled based on an anti-vector fight and the widespread use of antimalarial drugs, malaria epidemics could be explained in terms of three principal causes as developed below. The first cause involves the sudden increase in anopheline densities caused by prolonged periods of abnormal warm and rainy weather [123]. But in western Provence, this idea is backed up by the fact that epidemics have raged for more than five years while the weather had varied. The second cause, generally considered as one of the most probable, could be due to the arrival en bloc of a non-immune population into a malarious area or to the introduction of a number of infected individuals in an area where both the anopheline vectors and the conditions of transmission are present [123]. But in western Provence, this cause is irrelevant. The third cause, involves the introduction of new *Plasmodium* strains and/or the selection of new genetic variants. In past western Provence, the introduction of new *Plasmodium* strains or species may be responsible of epidemics; however, the data from the beginning of the 19^th^ century suggested that the return of soldiers from highly endemic areas does not seem to have an epidemiological impact, suggesting that indigenous vectors were most likely not efficient for the transmission of exotic *Plasmodium*. Therefore, the introduction of new strains was possible, but this had most likely not played a determining role in triggering epidemics. Study of the various, but very localized, epidemics linked to the digging of the canal from Arles to Bouc, which occurred after the great epidemic period, has underlined the extreme complexity of human malarial disease. Some epidemics were severe and very lethal, other relatively mild; some of the epidemics raged only among the foreign population, whereas others also affected the indigenous peoples. In addition, it seems that acquired immunity could be gained quickly but could be lost just as rapidly. This suggests that the critical factors could depend of the characteristics of the *Plasmodium* and on the level of acquired immunity. As *P. vivax* appears to have the potential to quickly acquire a high degree of genetic polymorphism [274], the hypothesis of the emergence of new genetic variants is highly plausible.

Statistical analyses have shown that years with epidemics tend to be warmer, especially during the early spring and in September and October, suggesting that relatively high temperatures would be useful in spring for the development of the first anopheline larvae and in autumn, thus allowing the extension of the period during which bites cause infection. Moreover, relatively high levels of precipitation were observed during December of the previous year, and even from November to September; this will also be a favouring factor (allowing to maintain a suitable level of liquid water and humidity for anopheline larvae and imagos respectively). In addition, cold winter temperatures and heat waves did not play a role in epidemics, suggesting a low impact of indoor malaria and *P. falciparum,* respectively. Multivariate analysis has shown that high temperatures (principally in early spring and in September and October) and the presence of an epidemic during the previous year are linked factors relating to rainfall. Moreover, mean temperatures during July and August could not be a determining factor governing epidemic malaria transmission but always theoretically allowed the outdoor sporogony of *P. vivax*.

The quick and definitive disappearance of great epidemics is the other puzzle of this study. In Europe, malaria was an endemic disease until after the end of World War II, and this disease is considered to have been eliminated using a mixture of countermeasures, such as vector control, disease treatment (principally extensive use of quinine and its derivatives), habitat modification and improvements in general living standards [3]. However, in some areas, malaria has declined, or even been eradicated, without deliberate countermeasures as in England [59,83,86] or Finland [30,101]. However, in Provence, all the various causes which have been proposed to explain the epidemic and endemic regressions of malaria, including drying of marshes, changes in cattle breeding practices and use of quinine are irrelevant. Moreover, during the studied period in this area, as in most of the European populations but unlike most of the other Mediterranean areas, the indigenous peoples should not have malaria-protective genetic traits.

Emergence, and therefore also the decline of malarial epidemic, depends to a great degree on the potential survival of mosquito vectors. Concerning western Provence, authors have suggested that the decline of both epidemic and endemic malaria was due to the decrease, for unknown reasons, of the number of mosquitoes during the 18^th^-19^th^ centuries [18], but this assertion seems inaccurate because during the epidemic period and also in the following mid-century period, the number of patients with intermittent fevers remained high, on one hand, and, on the other hand, the agricultural workers infected in fields were responsible for the spread of malaria in towns, so even in areas of relatively lower endemicity, vectors were always relatively efficient and abundant. However, the brutal collapse of the dynamics of two anopheline species observed in 2005 in Camargue without any identified cause [53] provided evidence that vector populations can quickly change, which might have an impact on the epidemic level. In addition, anopheline changes could be either quantitative (in terms of the number of individuals), qualitative (in terms of the species and/or of resistance to infection by *Plasmodium*), or also at the level of the feeding behaviour (zoophilic pattern versus anthropophilic of host selection). It has also hypothesized that the Provencal strains of *Plasmodium* had degenerated and that a set of factors would have led to the aging of malaria and to its disappearance [18], but at this stage, this hypothesis remains largely speculative. However, a hypothesis regarding a decrease of the virulence of the parasites is conceivable; indeed, analyses based on historical data could suggest that since the end of the 18^th^ century, the number and length of relapses in time would have decreased on average, which could have reduced the transmission rate, especially during the following year.

Statistical analyses have shown that two great climatic changes were observed after the breakpoint period. The rainfall in December months was lower, and previous analyses had shown that strong precipitation during this month significantly favoured epidemics. Moreover, a significant increase in the temperature fluctuations during the months of April, May, August and December was observed since the breakpoint and, interestingly, recent studies have shown that the key *Anopheles*/*Plasmodium*-related traits that combine to determine malaria transmission are all sensitive to variation in temperature [265-267]. To date, the fluctuations on temperature appeared to be a plausible explanation for the quick extinction of great malaria epidemics. These temperature variations, which could affect parasite infection, the rate of parasite development, and the essential elements of mosquito biology, could have disturbed the human-mosquito-parasite relationship and also could have slowly induced a reduction in the frequency and severity of malaria in western Provence. However, in view of the precarious ecological balance of malaria in a temperate environment, subtle interrelationships between several factors cannot be excluded.

Various data, including the long duration of some epidemics, could suggest that in spite of year-to-year climate changes, and therefore variations in anopheline density and the length of the transmission season, the condition of malaria transmission in Provence should be relatively stable. However, various elements demonstrate the specificity of malaria on the French Mediterranean continental coasts and also could suggest that despite the high level of endemicity, malaria could be unstable in this area. This detail may seem contradictory, for example, based on the only criterion of outdoor temperature. *P. vivax* malaria transmission was never interrupted, even during the coldest summers of the studied period in western Provence. However, the rapid extinction of great epidemics and the steep decline of endemicity without any deliberate countermeasures in less than three-quarters of a century argue in favour of this hypothesis, as in the other Mediterranean areas, only quinine and antimosquito pesticides were able to eradicate this plague. Malaria and its associated factors in western Provence exhibited either the characteristics of northern Europe or of southern Mediterranean Europe, which could explain its relative instability. In Provence, similarly to what has been observed in northern countries, the main parasite was *P. vivax* and the decline of endemic malaria was observed without deliberate countermeasures. Moreover, the indigenous population has no known genetic protective mutations. However, compared to the northern regions of Europe, the average temperature was higher and was always sufficiently favourable to always ensure transmission by outdoor mosquito bites. In addition, in Provence, there was probably little to no indoor transmission (outside periods where outdoor sporogony was possible) and, outside epidemic periods, the local *Plasmodium* strains seemed to have “tropical” characteristics: a weak latency period after the infectious bite followed by a rapid series of relapses. Therefore, the clinical signs were observed only for about three months, which could undermine the transmission of *Plasmodium* when the conditions were not optimal. Moreover, during epidemics, the high level of mortality and the clinical signs observed were similar to those of *P. falciparum* malaria. In addition, in Provence, the high temperatures and low rainfall in the summer allowed the labourers to sleep outdoors, and each year, this local habit resulted in the diffusion of *Plasmodium*, including its spread to healthier areas. Compared to most of the other Mediterranean areas, the mean temperature in Provence was generally lower, *P. falciparum* was certainly absent and the efficient dominant vector species of human malaria which was generally found in southern Europe; *Anopheles labranchiae*, *Anopheles sacharovi*, *Anopheles sergentii* and *Anopheles superpictus*[213] were likely to not be present (see for current distribution [275,276]). Whereas in stable condition, *P. falciparum* was frequently the most common parasite species, this case presents *P. vivax* in unstable conditions [75]; in western Provence, the lack of certainty about the presence of *P. falciparum*, the relatively low representation of *P. malariae* and the dominance of *P. vivax*, the most resilient *Plasmodium* species [111], suggest that in spite of the persistence of malaria for centuries, this area could be regarded as borderline for malaria parasites, ecologically speaking. The slowness of malaria penetration in this area, despite early colonization by the Romans and its geographical proximity to the north of the Italian Peninsula, also goes in this sense, even if the instability of malaria in Provence seems to contradict the fact that malaria has raged in this region for nearly 1,400 years [260]. This context just reflects the ambivalence of the malaria status of Provence. In future studies, it will be important to pay close attention to the complexities of this challenging subject particularly with regard to socio-economic variables, such as household size, which should be analysed if relevant data-sets could be generated.

## Competing interests

The authors declare that they have no competing interests.

## Authors’ contributions

ER and GP collected meteorological data, ER collected, from ancient publications and archives principally those of the Royal Society of Medicine and diaries (“livres de raison”), most of the malarial epidemics, GP collected various general data concerning Provence. MRC analysed meteorological data, carried out mathematical analysis. EF collected data on malaria in Provence, conceived the study and drafted the manuscript. All authors read and approved of the final manuscript.

## Supplementary Material

Additional file 1 **Climatic, epidemiologic and other miscellaneous data reported from 1745 to 1850.** Most of the data were collected from references [18,19,25,31,37-40,44,140,256] or references cited in the article. The letter “X” indicated that the event referred to in the column heading occurred during the year in question. For example, this letter was added when an epidemic or more were mentioned in one of the three areas. Concerning floods, when the episode occurred in November and/or December, it was postponed to the following year. Years with droughts had either been mentioned in historical records or had been observed by the meteorological services. Only floods observed in Arles and of a height greater than 4 meters are mentioned. Freezing of the Rhone indicated that the river was frozen or that there was floating ice. Click here for file

Additional file 2 **Filling of the gaps for temperature (a) and rainfall (b).** Mean monthly temperatures and total monthly rainfall underlined in pink corresponded to the averages for the known temperatures and precipitation. Click here for file

Additional file 3 **Populations and altitudes for the communes mentioned in the studied areas.** Communes: in France, a commune is the smallest administrative division of the territory. This subdivision dates only from the revolutionary period (1793) and generally corresponds to towns or villages with the surrounding land. For the communes located on the right bank of the Rhone River, which are not located in Provence, the department on which they depend is mentioned: Gard. Populations correspond to the census of 1793, n.k. = means “not known”. Altitudes: four altitudes are given: minimal, maximal, those of the town hall, which provides an approximate indication of the average altitude of inhabited areas and approximate mean altitudes. As commune areas can vary over the centuries, these values correspond to the current data. The mean elevation of a community corresponding to the sum of all the altitudes measured divided by the number of the latter; the accuracy of the measurement depends on the number of quoted points assessed. Almost all of the data are collected from [269,270]. Click here for file
