# Supplementary figures and images for: Analysis of the causes of spawning of large-scale, severe malarial epidemics and their rapid total extinction in western Provence, historically a highly endemic region of France (1745–1850)
Source: Malar J. 2014 Feb 28;13:72. doi: 10.1186/1475-2875-13-72 (PMC3939818; doi:10.1186/1475-2875-13-72)

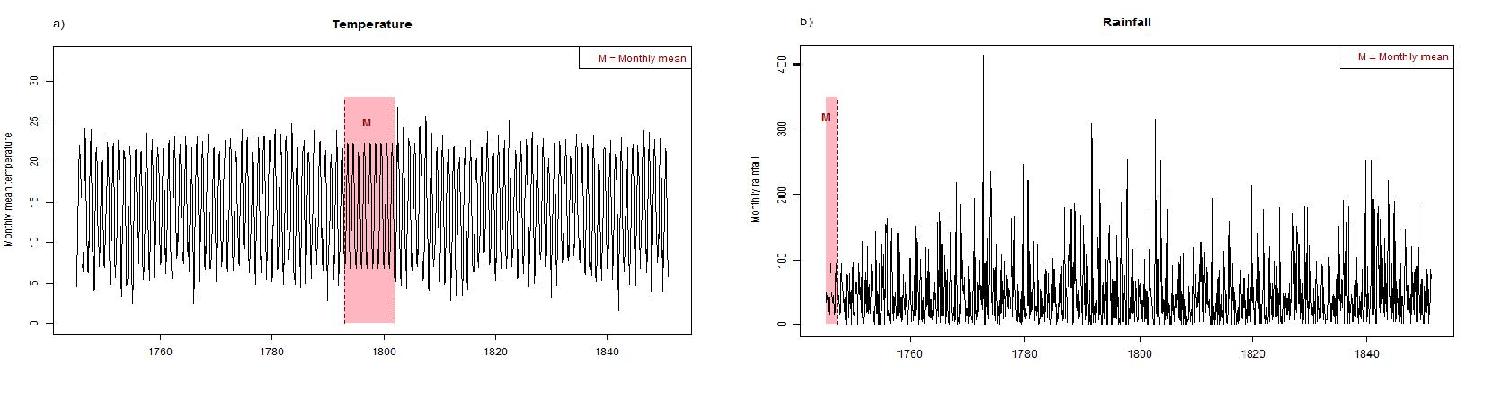

Supplement: Additional file 2 — Filling of the gaps for temperature (a) and rainfall (b). Mean monthly temperatures and total monthly rainfall underlined in pink corresponded to the averages for the known temperatures and precipitation. [file 1475-2875-13-72-S2.jpeg]
